# Genome Features of a New Double-Stranded RNA Helper Virus (LBCbarr) from Wine *Torulaspora delbrueckii* Killer Strains

**DOI:** 10.3390/ijms222413492

**Published:** 2021-12-16

**Authors:** Manuel Ramírez, Rocío Velázquez, Antonio López-Piñeiro, Alberto Martínez

**Affiliations:** 1 Departamento de Ciencias Biomédicas (Área de Microbiología), Facultad de Ciencias, Universidad de Extremadura, 06071 Badajoz, Spain; rociovelazquez1981@gmail.com (R.V.); amartinetp@alumnos.unex.es (A.M.); 2 Departamento de Biología Vegetal, Ecología y Ciencias de la Tierra, Facultad de Ciencias, Universidad de Extremadura, 06071 Badajoz, Spain; pineiro@unex.es

**Keywords:** *Torulaspora delbrueckii*, killer Kbarr, LBC helper virus, dsRNA genome, high-throughput sequencing

## Abstract

The killer phenotype of *Torulaspora delbrueckii* (Td) and *Saccharomyces cerevisiae* (Sc) is encoded in the genome of medium-size dsRNA viruses (V-M). Killer strains also contain a helper large size (4.6 kb) dsRNA virus (V-LA) which is required for maintenance and replication of V-M. Another large-size (4.6 kb) dsRNA virus (V-LBC), without known helper activity to date, may join V-LA and V-M in the same yeast. *T. delbrueckii* Kbarr1 killer strain contains the killer virus Mbarr1 in addition to two L viruses, TdV-LAbarr1 and TdV-LBCbarr1. In contrast, the *T. delbrueckii* Kbarr2 killer strain contains two M killer viruses (Mbarr1 and M1) and a LBC virus (TdV-LBCbarr2), which has helper capability to maintain both M viruses. The genomes of TdV-LBCbarr1 and TdV-LBCbarr2 were characterized by high-throughput sequencing (HTS). Both RNA genomes share sequence identity and similar organization with their ScV-LBC counterparts. They contain all conserved motifs required for translation, packaging, and replication of viral RNA. Their Gag-Pol amino-acid sequences also contain the features required for cap-snatching and RNA polymerase activity. However, some of these motifs and features are similar to those of LA viruses, which may explain that at least TdV-LBCbarr2 has a helper ability to maintain M killer viruses. Newly sequenced ScV-LBC genomes contained the same motifs and features previously found in LBC viruses, with the same genome location and secondary structure. Sequence comparison showed that LBC viruses belong to two clusters related to each species of yeast. No evidence for associated co-evolution of specific LBC with specific M virus was found. The presence of the same M1 virus in *S. cerevisiae* and *T. delbrueckii* raises the possibility of cross-species transmission of M viruses.

## 1. Introduction

Killer yeast strains can kill non-killer strains because they secrete proteins that are killer toxins. A certain type of killer yeast can also kill other types of killer strains that belong to the same yeast species. Some killer yeast strains can also kill yeasts (killer and non-killer) that belong to other species. Each killer yeast is immune to its own toxin and also to toxins secreted by other yeast strains with the same type of killer phenotype [1,2,3]. Best known killer phenotype of *Torulaspora delbrueckii* (Td) and *Saccharomyces cerevisiae* (Sc) is encoded by cytoplasmic medium-size dsRNA viruses (V-M). The killer yeast strains also contain a helper large-size (4.6 kb) dsRNA virus (V-LA) that is required for maintenance and replication of V-M. The helper V-LA provides the capsid and polymerase required for V-M maintenance. A specific LA virus may support different types of satellite M viruses but only one type of V-M has been discovered in each yeast killer type so far [1,2]. Another large-size (4.6 kb) dsRNA virus (V-LBC) may join V-LA and V-M in the cytoplasm of the same *S. cerevisiae* strain, although LBC viruses have not been described to date as having helper activity [3,4,5,6,7]. All of these viruses are assumed to be inherited in the cytoplasm from mother yeast to daughter bud, and spread horizontally between different yeasts by cell-cell mating or heterokaryon formation [8].

V-LA and V-LBC genomes encoded two proteins: the virion coat protein (Gag), and a fusion protein translated by a −1 ribosomal frameshifting mechanism (Gag-Pol) that contains the polymerase activities required for virus propagation. The slippery site for ribosomal frameshifting is “GGGUUUA” in V-LA, and “GGAUUUU” in V-LBC [7,9,10,11]. Some cis signals located in the 3′-terminal region of the (+)RNA are involved in its packaging and replication [2,7,9,10,11,12,13]. The (+)RNA of V-M contains some stem-loops that mimic those signals required for packaging and replication in V-LA, which allows M viruses to use the replication machinery of V-LA to propagate. Beside this, the signal for transcription initiation is located in the first 25 nucleotides of the 3′ end of (−)RNA, involving the terminal 3′CTTTTT motif in V-LA and 3′CTTAAA in V-LBC (5′GAAAAA and 5′GAATTT in the positive strand, respectively). V-M (+)RNA also mimic this V-LA motif in the 5′ end of its canonical sequence [3,5,12,14,15,16,17]. These functional motifs were primary characterized in ScV-LA [2]. Although LBC and LA viruses share similar genomic organization and modes of expression [7], the functional motifs of V-LBC are different than those of V-LA. These differences are supposed to be the reason why, in general, the LBC-dsRNA copy number is about 10–20% of that for LA dsRNA. Furthermore, this low amount of V-LBC could explain, at least in part, why this virus has no helper activity to maintain M viruses in *S. cerevisiae* [18,19].

As much as 19 V-LA isotypes have been found in *Saccharomyces sensu stricto* yeasts (*S. cerevisiae*, *S. paradoxus*, *S. kudriavzevii*, and *S. uvarum*). Genome sequences comparison allowed to define two clusters: the *S. cerevisiae* cluster that grouped most LA viruses from *S. cerevisiae* strains, and the *S. paradoxus* cluster that grouped most viruses of this yeast species and one from *S. uvarum*. Besides these two clusters, a third cluster was defined to include the only V-LA isolated from *T. delbrueckii* (TdV-LAbarr1), which was the most distant from the rest of the LA viruses [12]. A similar phylogenetic relationship for *Saccharomyces* LA viruses has also been reported elsewhere [4,6]. A similar genome comparison for V-LBC has not yet been performed as only the genomes of three *S. cerevisiae* viruses were available.

It has been suggested that some specific L and M viruses may co-evolve with each other in their natural environment. This was mainly based on the observed association of some V-L isotypes with specific M viruses in the same killer yeast [4,13]. This suggestion was not further confirmed in a subsequent study, although co-evolution of LA virus with a given yeast-host species may occur in a specific location or habitat [12].

The *T. delbrueckii* Kbarr1 killer strain (EX1180) contains the killer virus Mbarr1 plus two L viruses, TdV-LAbarr1 and TdV-LBCbarr1. The genome of TdV-LAbarr1 has recently been characterized [12], but the TdV-LBCbarr1 genome remains uncharacterized. It is unknown whether TdV-LBCbarr1 has helper activity due to the presence of TdV-LAbarr1 in the same yeast strain. On the contrary, *T. delbrueckii* Kbarr2 killer strain (EX1257) contains two M killer viruses, Mbarr1 and M1 [3], and we have recently discovered that this strain contains only a LBC virus (TdV-LBCbarr2) to maintain both M viruses. TdV-LBCbarr2 also remains uncharacterized. TdV-LBCbarr1 and TdV-LBCbarr2 are the first LBC viruses found in yeast different than *S. cerevisiae*, and Td EX1257 was the first yeast found to contain two M viruses at the same time.

The objective of this study was to determine the genome organization of the *T. delbrueckii* LBCbarr viruses as well as other LBC viruses from several *S. cerevisiae* strains isolated from the same geographical region. We addressed the following issues: (i) purification, sequencing, and characterization of two Td-LBCbarr viruses and five Sc-LBC viruses from different killer strains, and (ii) analysis of TdV-LBCbarr genome organization and its Gag-Pol ORF as compared with the rest of LBC viruses. We discuss the evolutionary relationship between these yeast viruses, as well as emphasize that both Td-LBCbarr viruses are not typical LBC viruses because they share some structural motifs with the (+)RNA of LA viruses.

## 2. Results

### 2.1. Analysis of the dsRNA Genomes from TdV-LBCbarr1 and TdV-LBCbarr2

Two different sequences were obtained from the L dsRNA (agarose electrophoresis band of 4.6 kb) present in the Kbarr1 EX1180 strain, one similar to that of LA virus genomes (named TdV-LAbarr1) that was previously characterized [12], and another sequence similar to that of LBC viruses (named TdV-LBCbarr1). However, only one sequence was obtained from the L dsRNA present in Kbarr2 EX1257 strain (named TdV-LBCbarr2). This strain contains two killer viruses, Mbarr1 and M1 [3], which indicates that TdV-LBCbarr2 is the helper virus required for replication and maintenance of both M viruses.

**Figure 1:**
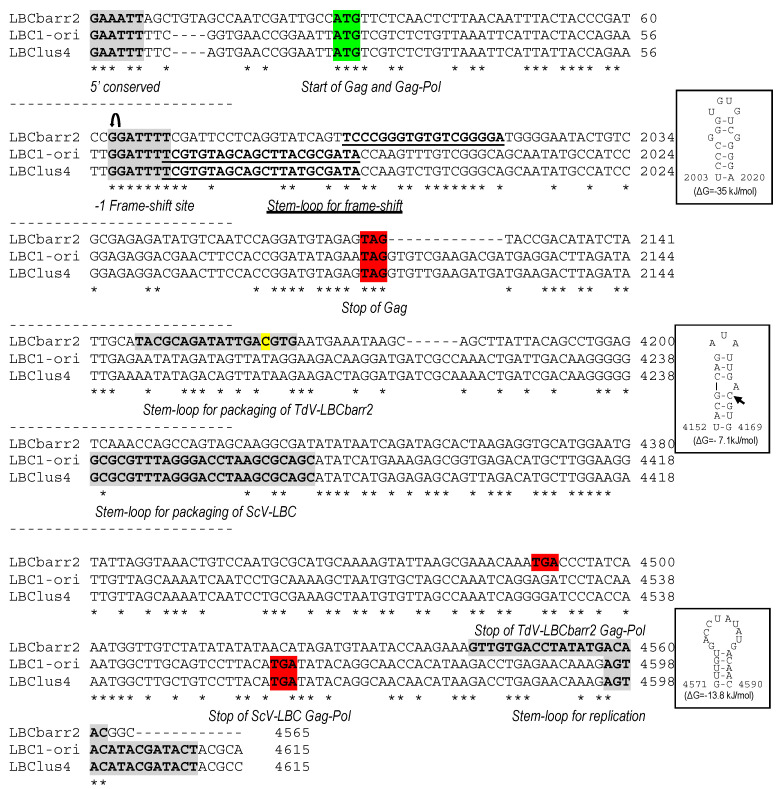
Partial multiple sequence alignment between ScV-LBC1-original, ScV-LBClus4, and TdV-LBCbarr2 (+) strand nucleotide sequences (cDNA). The full sequence alignment is presented in Supplemental Material (Figure S1). 5′GAA(A/T)TT conserved motif (5′ conserved), translation initiation (start of Gag and Gag-Pol), termination (stop of Gag or Gag-Pol) codons, ribosome frameshifting site (−1 frameshift site), frameshifting associated sequence (stem-loop for frameshift), packaging signal (stem-loop for packaging), and replication signal (stem-loop for replication) are indicated, shaded and/or underlined in the nucleotide sequence. Different residue in LBCbarr2 with respect to LBCbarr1 virus is yellow shaded. 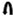, ribosomal frameshift. Asterisks (*) indicate identical nucleotide positions. The secondary structures of the putative cis signals for frameshifting, packaging, and replication of TdV-LBCbarr2 are displayed at the right of the sequence panel.

The complete sequence found for TdV-LBCbarr1 cDNA was 4763 nucleotides in length, and 5115 nt for TdV-LBCbarr2, which are slightly larger than the size estimated by agarose-gel electrophoresis (Table 1). Most of the sequence of both LBCbarr genomes (4565 nt central stretch) showed about 52% nucleotide identity with the previously known ScV-LBC1-original and ScV-LBClus4 genomes (Figure 1, Figures S1 and S3), while it only shared 39% identity with *Saccharomyces* and *Torulaspora* LA genomes. We considered this central stretch as the canonical sequence of LBCbarr genomes, and sequences upstream and downstream as 5′- and 3′-extra sequences, respectively; as it was previously considered for V-LA and V-M genomes [3,12]. The canonical sequences of TdV-LBCbarr1 and TdV-LBCbarr2 genomes share great identity (94%), which means that both are the same virus with some nt-sequence differences. Therefore, we mainly focused on the TdV-LBCbarr2 sequence for genome comparison with other L viruses.

TdV-LBCbarr2 canonical genome length (4565 bp) is slightly shorter than that of ScV-LA (4580 bp), TdV-LA (4591 bp), and ScV-LBC (4615 bp). Its genome organization is similar to that described for ScV-LA1-original, containing two ORFs [11]. The first ORF (from nt 28 to nt 2124) belongs to the coat (Gag) protein of the virion, and the second ORF (from nt 2157 to nt 4489) belongs to the viral RNA-dependent RNA polymerase (RdRp). This polymerase is expected to be synthetized as a Gag-Pol fusion protein by a −1 ribosomal frameshift at the conserved frameshifting site (or slippery site) located upstream of the Gag ORF stop codon (1977GGAUUUU1983) (Figure 1 and Figure S1), as previously described for ScV-LA1-original and ScV-LBC1-original [4,7,11,12]. These different ORF assignments were done by also considering the amino-acid sequence homology of the Gag-Pol fusion protein of both TdV-LBCbarr to those of L viruses from *Saccharomyces* (see below).

**Figure 2:**
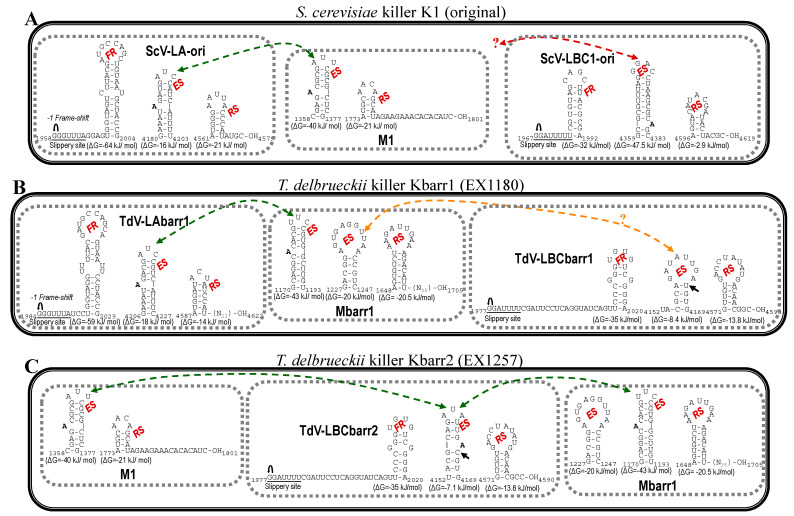
Secondary structure representations of signals for frameshifting, encapsidation, and replication present in the (+)RNA strand of L and M viruses from killer yeasts: (**A**) *S. cerevisiae* K1 (original), (**B**) *T. delbrueckii* Kbarr1 (EX1180), and (**C**) *T. delbrueckii* Kbarr2 (EX1257). FR, frameshifting region. ES, encapsidation signal. RS, replication signal. 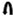, ribosomal frameshift. Black arrow indicates the single nucleotide changes between ES of LBCbarr1 and LBCbarr2. Green dot-line arrow indicates equivalent ES for L and M viruses in the same yeast. Orange dot-line arrow indicates possible equivalent ES for LBCbarr1 and Mbarr1 in Kbarr1 EX1180 yeast. Red dot-line arrow indicates unknown equivalent ES in M viruses for the ES of ScV-LBC1-original.

There are other important regions in TdV-LBCbarr genomes that are similar to those highly conserved in ScV-LA and ScV-LBC genomes [4,6,12,13,16]: (i) a 18-nt stem-loop region involved in frameshifting (2003TCCCGGGTGTGTCGGGGA2020), that is located 19 nt downstream from the slippery site; (ii) a 18-nt stem-loop (4152TACGCAGATATTGACGTG4169), that is located 174 nt downstream from the RdRp domain and is responsible for binding to and packaging of the (+)RNA strand of L viruses; and (iii) a 20-nt stem-loop (4543GTTGTGACCTATATGACAAC4562), that is located 3 nt upstream from the 3′ end and is responsible for RNA replication (Figure 1 and Figure S1). These motifs are found in both TdV-LBCbarr genomes in equivalent positions with respect to the genome sequence of ScV-LA1-original, which is the best known among the yeast L viruses [11]. In ScV-LA1-original, the stem-loop for frameshifting is located 4 nt downstream from the slippery site, the stem-loop for (+)RNA packaging is 191 nt downstream from RdRp domain, and the stem-loop for RNA replication is 2–4 nt upstream from the 3′ end. These locations are very similar to that found in both TdV-LBCbarr genomes: 19 nt, 174 nt, and 3 nt, respectively. The only difference between both TdV-LBCbarr genomes is one nt change in the 18-nt stem-loop required for virus packaging, that is 4152TACGCAGATATTGATGTG4169 in LBCbarr1 and 4152TACGCAGATATTGACGTG4169 in LBCbarr2. As a consequence, only the secondary structure of LBCbarr2 stem-loop is similar to that found in ScV-LA1-original in the sense of this stem-loop shows a critical “A” residue protruding from the stem in both cases [14,20]. However, this “A” is protruding from the 3′ side of the stem in TdV-LBCbarr2, as in ScV-LBC1-original, instead from the 5′ side of the stem as found in ScV-LA1-original and TdV-LAbarr1 (Figure 2).

Although these three stem-loops are also assumed to be present in the genome of *S. cerevisiae* LBC viruses, they are not easily identified in equivalent positions with respect to the nucleotide sequence of ScV-LA1-original. Some stem-loops have been suggested as required for frameshifting, RNA packaging, and RNA replication of ScV-LBC1-original (Figure 1 and Figure S1) [4,21]. We found these proposed signals in all the newly sequenced ScV-LBC genomes, in the same location. As previously described, the stem-loop proposed for frameshifting in ScV-LBC genomes is overlapping 1 nt with the ribosome slippery site [4], the stem-loop for (+)RNA packaging is 349 nt downstream from RdRp domain (which is 158 nt downstream from the expected location in LA viruses [12,13], and the two possible stem-loops for RNA replication (separated 9 nt of distance between them, and located 4–6 nt upstream from the 3′ end [21]) are weak stem-loop structures (ΔG = −7.5 and −2.9 kJ/mol, respectively) (Figure 1, Figures S1 and S2).

TdV-LBCbarr genomes also contain the 5′ AU-rich regions of L and M dsRNA viruses that facilitate “melting” of the molecule and the access of the RNA polymerase to the (−)RNA template for conservative transcription [3,5,17,22]. In this case, this motif (5′GAAATT) is more similar to that found in ScV-LBC genomes (5′GAATTT) than to that found in ScV-LA genomes (5′GAAAAA) [12].

### 2.2. Analysis of the Gag-Pol Sequences from TdV-LBCbarr1 and TdV-LBCbarr2

**Figure 3:**
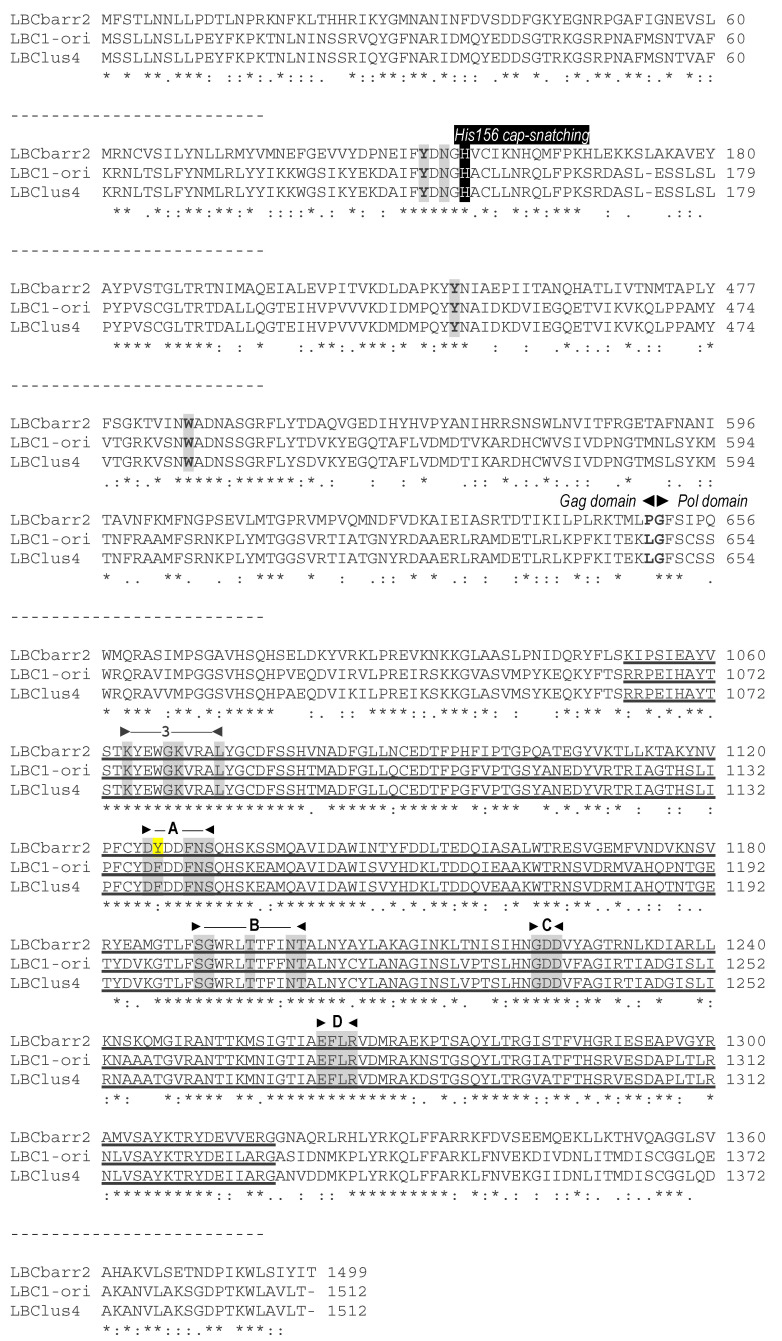
Comparison between partial amino-acid sequences of Gag-Pol encoded by ScV-LBC1-original, ScV-LBClus4, and TdV-LBCbarr2 genomes. The full sequence alignment is presented in Supplemental Material (Figure S2). The separation between Gag and Pol is indicated (Gag◄►Pol). The H156 residue required for 5′cap-snatching is black shaded. The four crucial residues for cap recognition (Tyr-152, Asn-154, Tyr-452 and Trp-545) are grey shaded. The highly conserved central third of Pol (RdRp domain) is underlined, the five consensus motifs (3, A, B, C, and D) conserved in RNA-dependent RNA polymerases from Totiviruses are indicated above the sequence, and the conserved amino acids for each motif are grey shaded. Different residue in TdV-LBCbarr with respect to ScV-LBC is yellow shaded. Asterisks (*) indicate identical amino acids; colons (:) and single dots (.) indicate conserved and semi-conserved amino acids, respectively.

**Figure 4:**
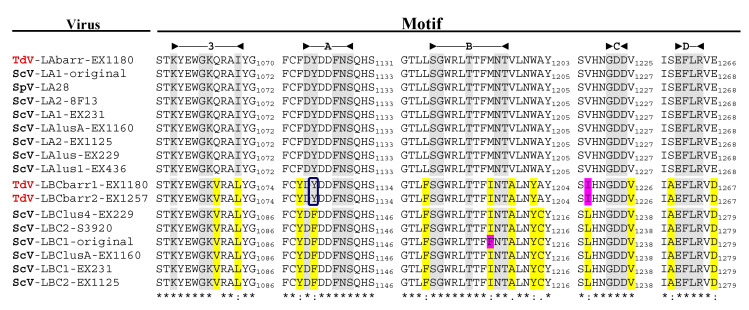
Conserved motifs in the Gag-Pol RdRp domain of L viruses from *S. cerevisiae* and *T. delbrueclii*. The extent of each domain is indicated in the top of the figure. The last amino acid of the stretch containing each motif is indicated to the right of the sequence. Asterisks (*) indicate identical amino acids; colons (:) and single dots (.) indicate conserved and semi-conserved amino acids, respectively. Conserved identical residues in all virus motifs are grey shaded. Different residues in LBC with respect to LA viruses are yellow shaded. Different residues for TdV-LBCbarr and ScV-LBC1-original are pink shaded. “Y” residue in motif A of TdV-LBCbarr that is coincident with LA viruses is indicated inside a square.

**Figure 5:**
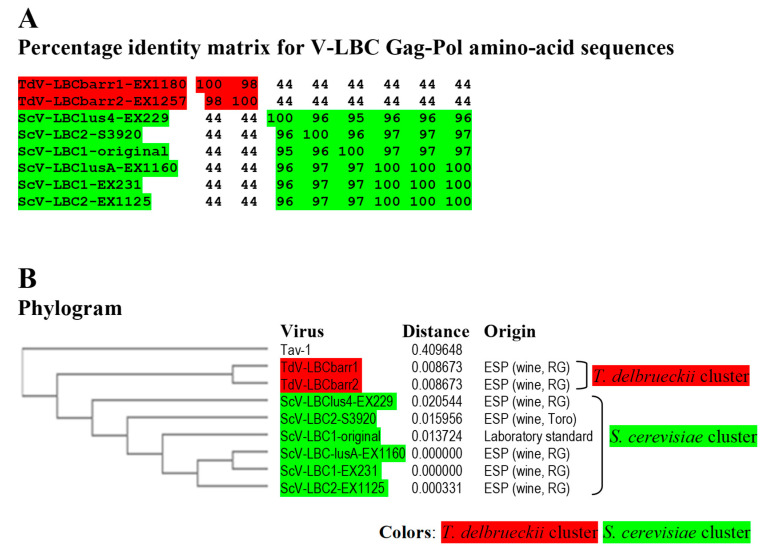
Phylogenetic relationship of yeast LBC viruses. (**A**) Percentage identity matrix between the complete amino acid sequences of the Gag-Pol proteins of LBC viruses. Each identity value was rounded to the nearest whole number. (**B**) Phylogram with evolutionary distances (given by the MUSCLE program) and geographical location at which each killer yeast strain was isolated. RG, Ribera del Guadiana region in Extremadura. ESP, Spain. Viruses included in each of the two main clusters, as well as their identity values, are shaded in red or green. The Tuber aestivum (Tav-1) totivirus was used as outgroup (GenBank accession number HQ158596.1).

No relevant identity was found between the putative Gag-Pol amino acid sequences of both TdV-LBCbarr and those of LA viruses (24–24.8%), similarly to that found between Gag-Pol of ScV-LBC1-original and ScV-LA1-original (24.6%). TdV-LBCbarr Gag-Pol sequences showed 44% global identity with that of ScV-LBC1-original (Figure 3, Figure 5 and Figure S2). Despite this modest identity, both LBCbarr Gag-Pol sequences contain most of the key motifs for Gag and Pol functions previously found in *Saccharomyces* LBC viruses. This identity is much lower than that found among Gag-Pol sequences of known *S. cerevisiae* LBC-viruses, which is greater than 95% ([4] and see below). This modest identity increases to 64–66% when comparing only the highly-conserved RdRp-domain of TdV-LBCbarr and ScV-LBC polymerases. This domain contains the five motifs (3, A, B, C, and D) conserved among RdRps, which are essential for RNA polymerase activity [23,24]. The most conserved amino acids of these five motifs are identical in all RdRps of yeast L viruses except one amino acid in motif A, which is “DYDDFNS” in all known *Saccharomyces* and *T. delbrueckii* LA viruses, and “DFDDFNS” in all *S. cerevisiae* LBC viruses. Surprisingly, motif A of both TdV-LBCbarr polymerases is identical to that found in LA viruses. However, surrounding residues of these five conserved amino acids of LBCbarr polymerases are in most cases coincident with those of *S. cerevisiae* LBC viruses (Figure 4).

The identity between Gag sequences of TdV-LBCbarr and those of ScV-LBC viruses also is low, about 24–37.8%. However, the Gag His154 residue of ScV-LA1-original (that is His155 in ScV-LBC1-original, and His156 in TdV-LBCbarr) required for the cap-snatching mechanism in the virion [25], and the four putative crucial residues for cap recognition (Tyr-150, Asp-152, Tyr-452, and Tyr-538 in ScV-LA1-original; that are Tyr-152, Asp-154, Tyr-449 and Trp-543 in ScV-LBC1-original [26]) are also present in the Gag protein of both LBCbarr viruses (Tyr-152, Asn-154, Tyr-452 and Trp-545; Figure 3 and Figure S2). In addition to the slightly different location for each residue in the Gag sequence, the main difference among these viruses is that the forth residue is Trp in LBC1-original and LBCbarr viruses, while it is Tyr in ScV-LA1-original. The same difference was found when comparing all known LA and LBC viruses from *Saccharomyces* and *T. delbrueckii* yeasts (data not shown).

### 2.3. Comparison of TdV-LBCbarr with LBC Viruses from Saccharomyces Yeasts

The dsRNA and Gag-Pol sequences of both TdV-LBCbarr were compared with their counterparts from *S. cerevisiae* to analyze their phylogenetic relationship. The genomic sequence of ScV-LBC1-original, ScV-LBClus-EX229, and ScV-LBC2-S3920 were already known (Table 1 and Table 2). The genomes of ScV-LBC1 from strain EX231, ScV-LBC2 from EX1125, and ScV-LAlusA from EX1160 were *de novo* sequenced by HTS techniques for this study. ScV-LBClus4 from EX229, previously sequenced by Sanger procedure and named ScV-L-BC-lus [4], was also de novo sequenced to assess the accuracy of our HTS procedure. Sequences obtained by both procedures share 99.9% identity. Only six nucleotide changes were found in the genome of ScV-LBClus4 with respect to ScV-L-BC-lus: C548A, T704C, T2667G, G2668C, G2669T, and G2671A. As nucleotides of ScV-LBClus4 (HTS) in these positions coincided best with the rest of the *S. cerevisiae* LBC viruses, this sequence was the one used for further comparison.

Identity among Sc-LBC viruses was similar to that previously found [4], above 95% for amino acid sequences. Two clusters were found to include all LBC viruses: S. cerevisiae cluster, in which ScV-LBClusA-EX1160, ScV-LBC1-EX231 and ScV-LBC2-EX1125 isolated from Extremadura seem to be the same virus (99.9–100% identity of Gag-Pol); and T. delbrueckii cluster grouping TdV-LBCbarr1-EX1180 and TdV-LBCbarr2-EX1257, that were also isolated from Extremadura, and also appear to be the same virus (98.3%). Viruses from different clusters share a fairly low identity rate (43.5–44.2%), despite that most viruses were isolated from the same region. Given the high similarity of ScV-LBClus4-EX229, ScV-LBC2-S3920 and ScV-LBC1-original with the rest Sc-LBC virus (95–100%), they appear to be just variants of the same virus (Figure 5). It is noteworthy that the same LBC virus was found can coexist with different killer M viruses in different yeast strains (Table 1).

## 3. Discussion

### 3.1. Analysis of TdV-LBCbarr Genomes

The average nucleotide identity between both T. delbrueckii LBCbarr viruses and S. cerevisiae LBC viruses (52%) was lower than that found between LA viruses of the same yeast species (60%) [12]. Moreover, this nucleotide identity was much lower than that found between Sc-LBC viruses (89–100%). This can be expected because these two yeast species are usually found in different ecological niches [12,28,29]. This finding raises the possibility that LBCbarr viruses are a new type of virus different from the already known LA and LBC viruses. Notwithstanding this, the organization of both TdV-LBCbarr genomes is similar to that of ScV-LA and ScV-LBC. They contain the same two ORFs, Gag and Gag-Pol. However, while TdV-LA and ScV-LA share 87.5–100% identity in some regions considered important for the virus replication (such as the frameshifting region required for translation of Gag-Pol fusion protein, or the virus packaging signal), TdV-LBC and ScV-LBC only share the ribosome slippery site located upstream of the Gag ORF stop codon (GGAUUUU). Interestingly, the three stem-loops involved in frameshifting, RNA binding and packaging, and RNA replication of TdV-LBCbarr genomes resemble those secondary structures found in similar positions in ScV-LA genomes, especially those of TdV-LBCbarr2 (Figure 2). This may explain why Sc-LA and Td-LBCbarr viruses have capability to maintain killer M viruses; whereas Sc-LBC viruses that have these stem-loops located in other positions do not have not this capability. TdV-LBCbarr2 contains a stem-loop for packaging and encapsidation (ES signal) similar to the ES of S. cerevisiae LA and M1 viruses, and also similar to the ES of TdV-Mbarr1. This may explain why TdV-LBCbarr2 has capability to maintain both M1 and Mbarr1 viruses in the same cell. However, a difference of only one nucleotide in LBCbarr1 ES with respect to LBCbarr2 ES (C4166T) changes its secondary structure, making LBCbarr1 probably unable to maintain the M1 virus. Optionally, the ES secondary structure of LBCbarr1 is similar to a newly identified possible ES in the Mbarr1 genome, raising the possibility that LBCbarr1 may maintain Mbarr1 at the same time that TdV-LAbarr1 does (Figure 2). I.e., Mbarr1 could be maintained by two different helper viruses in EX1180 strain, Td-LAbarr1 and Td-LBCbarr1.

We confirmed the presence of all motifs and features previously proposed as required for viral replication of ScV-LBC1-original in all the newly sequenced S. cerevisiae LBC genomes; and the particular location of the stem-loop proposed for frameshifting, that overlaps 1 nt with the ribosome slippery site [4] (Figure 2). The ribosome frame shift in L viruses happens with low frequency, in about 1% of ribosomes [30]. The overlapping of the slippery site and the frameshifting stem-loop may imply a steric effect that decreases the frame shift frequency, reducing the amount of Cap-Pol required for viral replication. This may explain, at least in part, the low relative amount of LBC virus with respect to LA virus in S. cerevisiae [9,31]. In addition to this, the genome location and secondary structure of the stem-loops involved in viral replication, that is different to those of Sc-LA and Td-LBCbarr viruses, may account for the inability of Sc-LBC viruses to behave as helper virus to maintain killer M viruses.

### 3.2. Analysis of TdV-LBCbarr Gag-Pol Sequences

Gag-Pol sequence of LBCbarr contained most of the key motifs for Gag and Pol functions previously described in ScV-LBC1-original, as well as similar surrounding residues. However, motif A of RdRp domain is identical in TdV-LBCbarr and LA viruses. This motif A is highly conserved in viruses of the family Totiviridae, and is clearly of functional importance for the polymerase. Moreover, in addition, motifs 3, B, C and D, is required for the support of M1 killer virus by ScV-LA1-original [24]. This finding, together with the similarity found between relevant motifs in the genome of LBCbarr and LA viruses (stem-loops for frameshifting, RNA packaging, and RNA replication), again suggests that TdV-LBCbarr (but not ScV-LBC) may have similar capability than ScV-LA for replication and helping other satellite M killer viruses to replicate in the same yeast cell. I.e., Td-LBCbarr viruses, and especially TdV-LBCbarr2 do not seem to be typical LBC viruses because they share several functional motifs with LA viruses. This contrasts with what was previously found for TdV-LAbarr1, which appears to be a typical LA virus similar to those already found in S. cerevisiae, although it belongs to a different genus of yeast [12].

### 3.3. Phylogenetic Relationship and Transmission of LBC Viruses

Only two clusters were found when comparing the nucleotide or amino acid sequences of all LBC viruses, both directly related to the host yeast species of the viruses. The S. cerevisiae cluster includes all ScV-LBC isolated from Extremadura, as well as two viruses isolated from elsewhere (ScV-LBC2-S3920 and ScV-LBC1-original); and the T. delbrueckii cluster includes the only two TdV-LBCbarr available, which are also isolated from of Extremadura although from locations sited about 70 km away. It appears that genome analysis of new LBC viruses, isolated from different habitats and locations in the world, is required in order to acquire comprehensive knowledge of the phylogenetic relationship of these viruses. Despite this, we did achieve some interesting findings, such as: (i) the same LBC virus can coexist with different killer M viruses in different S. cerevisiae strains, (ii) LBCbarr2 can support two different viruses (M1 and Mbarr1) in the same T. delbrueckii strain, and (iii) the same M virus can be supported by different L viruses (M1 by LA in S. cerevisiae or by LBCbarr2 in T. delbrueckii). This indicates that there is not an association or co-evolution of specific LBC with specific M viruses, contrary to that previously suggested [4]. Similar results were previously found for LA and M viruses [12]. Furthermore, the presence of the same M virus (M1) in different yeast species (S. cerevisiae and T. delbrueckii) raises the possibility of cross-species transmission among different yeast species, which eventually may coincide in the same habitat as wine fermentation [4,12]. However, the contrary may be suggested for LBC viruses, since Td-LBCbarr viruses are the most distant from the rest of Sc-LBC viruses (44% Gag-Pol identity), even from those of S. cerevisiae strains isolated from the same location (Figure 5B). Besides this, as previously suggested for LA viruses [12], a co-evolution of a specific LBC virus with its specific yeast host can be hypothesized.

The percentage of identity between Gag sequences of T. delbrueckii and Saccharomyces LBC viruses was lower than that found for full Gag-Pol or RdRp-domain sequences. These results are similar to that previously found for comparison between T. delbrueckii and Saccharomyces LA viruses. As a consequence, a comparison of Gag amino-acid sequence can be considered the best option for grouping LA and LBC viruses that are closely related [12].

## 4. Materials and Methods

### 4.1. Yeast Strains and Culture Media

T. delbrueckii killer Kbarr1(EX1180) and Kbarr2 (EX1257) yeasts are prototrophic strains isolated from the spontaneous fermentation of grapes from vines located in the Albarregas river valley of Mérida and southern Badajoz (both in Extremadura, southwestern Spain), respectively [3,15]. S. cerevisiae killer strains EX231, EX1125, EX229, EX231, and EX1160 were also isolated from wine spontaneous fermentations in close locations of the Ribera del Guadiana region in Extremadura. These strains show different mtDNA RFLP profiles and contain different M dsRNA isotypes [3,32]. All these yeasts are also prototrophic strains. Table 3 summarizes the yeasts used in this study. The killer phenotype and presence of viral dsRNA (L and M) in these yeasts was analyzed previously [3,12,15]. Standard culture media were used for yeast growth [33].

### 4.2. Purification of V-LBC dsRNA from Killer Yeasts

Nucleic acid Samples from killer yeast strains were obtained as described previously [12,34]. CF-11 cellulose chromatography was used to obtain the dsRNA from each yeast strain [35]. Agarose (1%) gel electrophoresis was used for separation of L and M dsRNA of each sample. The slower-moving dsRNA band (4.6 kb) was cut out of the gel and purified with RNaid^®^ Kit (MP Biomedicals, LLC, Illkrich, France). This procedure was repeated for each L virus to obtain at least 20 μg of dsRNA.

### 4.3. Preparation of cDNA Libraries from Purified V-LBC dsRNA and DNA Sequencing

Library preparation of cDNA and high-throughput sequencing (HTS) were done at the Unidad de Genómica Cantoblanco (Fundación Parque Científico de Madrid, Spain) as described previously [12]. Very briefly, the first strand of cDNA was synthesized using random primers dTVN and dABN (from Isogen Life Science, De Meern, The Netherlands) and SuperScriptIII retrotranscriptase. Thereafter, the second cDNA strand synthesis, end repair, 3′-end adenylation, and ligation of the TruSeq adaptors were done (Illumina). The adaptor oligonucleotides include signals for further amplification and sequencing, and also short sequences referred to as indices which allow multiplexing in the sequencing run. An enrichment procedure by PCR was performed to amplify the library, ensuring that all the molecules in the library included the desired adaptors at both ends. The final libraries were denatured prior to seeding on a flow cell, and sequenced on a MiSeq instrument using 2 × 80–2 × 150 sequencing runs.

### 4.4. Sequence Assembly of Virus Genomes

The analysis and assembling of cDNA sequences were done by the company Biotechvana (Technological Park of Valencia, Spain) as previously described [12]. SOAP deNOVO2 [36] was used to obtain a de novo assembly based on two Illumina libraries for each virus, trying multiple assembly attempts with scaffolding and insert size of 200, and varying the Kmer value (being 47 the most effective). Contigs shorter than 300 nucleotides were removed from the contig file. The remaining contigs were used as input to the NR database of the NCBI via the BLASTX search protocol [37] implemented in the GPRO 1.1 software [38]. Highly significant similarity was found between several contigs/scaffolds and some known viral RNA sequences (LA, LBC, and others) or host transcripts. Supposed contaminating sequences non-homologous to previously known LBC genomes were filtered from the assembly. Each virus was sequenced at least three times using independent samples and different dates during a period of several years. Full coverage of the canonical genome sequence was obtained at least twice for each virus, and 100% identity was found for all sequences obtained from the same yeast strain. Only full coverage sequences were used for comparison of viral genomes from different yeasts.

### 4.5. Sequence Analysis Tools

The sequence identity and phylogenetic relationship (phylogram) among LA genomes were obtained by the ClustalW(2.1) program for comparing nucleotide sequences [39], and MUSCLE(3.8) program for comparing amino-acid sequences [40]. The MFOLD program (http://unafold.rna.albany.edu/?q=mfold/RNA-Folding-Form, accessed on 10 November 2021) was used to predict the folding of ssRNA [41]. The parameters used were: folding temperature fixed at 37 °C; ionic conditions, 1M NaCl, no divalent ions; percent suboptimality number, 5; upper bound on the number of computed foldings, 50; maximum interior/bulge loop size, 30; maximum asymmetry of an interior/bulge loop, 30; maximum distance between paired bases, no limit.

### 4.6. Nucleotide Sequence Accession Numbers

ScV-LBClus genome from EX229 was previously analyzed by traditional techniques of cloning and sequencing [4], as well as those of ScV-LBC1-original [7], ScV-LBC2-S3920 [4], ScV-LA1-original [11], ScV-LA2-8F13 [4], SpV-LA28 [27]. TdV-LAbarr1-EX1180, ScV-LA1-EX231, ScV-LAlus4-EX229, ScV-LAlus1-EX436, ScV-LAlusA-EX1160, and ScV-LA2-EX1125 were previously sequences by HTS techniques [12], The GenBank accession number of these previously known genome sequences of L viruses are shown in Table 2.

The cDNA nucleotide sequence and amino-acid sequence of the Gag-Pol protein of newly sequenced (HTS) LBC viruses appear in NCBI/GenBank under the following accession numbers: TdV-LBCbarr1 from strain EX1180, (OL469171); TdV-LBCbarr2 from EX1257, (OL469172); ScV-LBC1 from EX231, (OL469175); ScV-LBC2 from EX1125, (OL469176); and ScV-LBClusA from EX1160, (OL469174). Although ScV-LBClus genome from EX229 was sequenced previously, it was de novo sequenced by HTS techniques for this study, and renamed as ScV-LBClus4 because it comes from a killer Klus-4 type strain (accession number: (OL469173).

## 5. Conclusions

Td-LBCbarr viruses share some genome features (such as nucleotide sequence identity, ribosome frame-shift slippery site, and conserved 5′GAAATT) and most Gag-Pol functional motifs (involved in cap snatching and RdRp activity) with Sc-LBC viruses. However, TdV-LBCbarr2 resembles LA viruses in the genomic signals related to RNA encapsidation and replication, and in the motif A of RdRp domain; which provide it with a helper capability to maintain up to two M viruses in the same yeast cell. This raises the possibility of LBCbarr viruses being a new type of virus different to the already known LA and LBC viruses. The discovering of new viruses similar to LBCbarr will be required to examine this question. The co-evolution of the LBC and M viruses seems unlikely, although the co-evolution of LBCbarr viruses with a given yeast host may occur in a specific habitat or location.

## Figures and Tables

**Table 1:** Characteristics of the dsRNA LBC-virus genomes sequenced by HTS.

Virus	Previous Sequenced Length (bp)/Yeast Strain	Newly Analysed Yeast Strain	Killer Phenotype/dsRNA Isotype	Sequenced Length (bp) (Canonical)
LBC1	4615/*Sc* 299 (LBC1-original)	*Sc* EX231	K1/M1-1	4971 (4615)
LBC2	4614/*Sc* S3920	*Sc* EX1125	K2/M2-4	4722 (4615)
LBClus	4614/*Sc* EX229	*Sc* EX229	Klus/Mlus4	4839 (4615)
		*Sc* EX1160	Klus/MlusA	4661 (4615)
LBCbarr-1	Unknown/*Td* EX1180	*Td* EX1180	Kbarr1/LBCbarr1	4763 (4565)
LBCbarr-2	Unknown/*Td* EX1257	*Td* EX1257	Kbarr2/LBCbarr2	5115 (4565)

*Sc*, *Saccharomyces cerevisiae*. *Td*, *Torulaspora delbrueckii*.

**Table 2:** Previously known nucleotide sequence of LBC and LA viruses used in this study.

Virus	Accession Number	Reference/Comment
ScV-LBC1-original	U01060.1	Previously [7] known as ScV-La or L-B-C. Renamed in this study to distinguish it from other LBC viruses from different K1 killer yeasts
ScV-LBClus4-EX229	KT784813.1	[4]
ScV-LBC2-S3920	KX906605.1	[4]
TdV-LAbarr1-EX1180	MW174763	[12]
ScV-LA1-original	J04692.1	Previously [11] known as ScV-LA. Renamed in this study to distinguish it from other LA viruses from different K1 killer yeast strains
ScV-LA1-EX231	MW174760	[12]
ScV-LAlus-EX229	JN819511.1	[13]
ScV-LAlus4-EX229	MW174758	[12]
ScV-LAlus1-EX436	MW174761	[12]
ScV-LAlusA-EX1160	MW174762	[12]
ScV-LA2-S3920	KC677754.1	[4]
ScV-LA2-EX1125	MW174759	[12]
SpV-LA28	KU845301.2	Formerly [27] assigned to *S. cerevisiae* but recently re-assigned to *S. paradoxus* [6]

Sc, *Saccharomyces cerevisiae*. Td, *Torulaspora delbrueckii*. Sp, *Saccharomyces paradoxus*.

**Table 3:** Yeasts used in this study.

Strain	Genotype [Relevant Phenotype]	Origin, Date, Grape Variety, Geographical Location in Extremadura (Spain)
*Td* EX1180	*wt* LAbarr1 LBCbarr1 Mbarr1 [Kbarr1^+^]	Wine, 2006, Cayetana, Mérida
*Td* EX1257	*wt* LAbarr2 LBCbarr2 Mbarr1 M1 [Kbarr2^+^]	Wine, 2006, Tempranillo, Badajoz
*Sc* EX231	*wt MAT a/*α *HO/HO* LA1 LBC1 M1-1 [K1^+^]	Wine, 2003, Macabeo, Guadajira
*Sc* EX1125	*wt MAT a/*α *HO/HO* LA2 LBC1 M2-4 [K2^+^]	Wine, 2005, Moscatel, La Albuera
*Sc* EX229	*wt MAT a/α HO/HO* LAlus4 LBClus4 Mlus4 [Klus^+^]	Wine, 2003, Macabeo, Guadajira
*Sc* EX1160	*wt MAT a/*α *HO/HO* LAlusA LBClusA MlusA MlusB MlusC [Klus^+^]	Wine, 2005, Moscatel, La Albuera

*Sc*, *Saccharomyces cerevisiae*. *Td*, *Torulaspora delbrueckii*.

## Data Availability

Not applicable.

## Supplementary Materials

The following are available online at https://www.mdpi.com/article/10.3390/ijms222413492/s1.
