# Supplementary material for: Genome Features of a New Double-Stranded RNA Helper Virus (LBCbarr) from Wine Torulaspora delbrueckii Killer Strains
Source: Int J Mol Sci. 2021 Dec 16;22(24):13492. doi: 10.3390/ijms222413492 (PMC8709356; doi:10.3390/ijms222413492)
Supplement: Supplementary file 1 [file ijms-22-13492-s001.zip › ijms-1481496-supplementary.pdf]

Supplementary Materials

1.1 Supplementary Figures

```
LBCbarr2 GAAATTAGCTGTAGCCAATCGATTGCCATGTTCTCAACTCTTAACAATTTACTACCCGAT 60
LBC-ori  GAATTTTTC----GGTGAACCGGAATTATGTCGTCTCTGTTAAATTCATTACTACCAGAA 56
LBClus4  GAATTTTTC----AGTGAACCGGAATTATGTCGTCTCTGTTAAATTCATTATTACCAGAA 56
         *** **  *        * *       ****  **     * **    *** **** **
         5' conserved          Start of Gag and Gag-Pol
LBCbarr2 ACTTTAAACCCTCGTAAGAATTTCAAACTTACTCACCATAGAATTAAATATGGTATGAAT 120
LBC-ori  TATTTTAAACCTAAAACTAATTTGAATATCAACTCTTCTAGGGTCCAATATGGCTTTAAT 116
LBClus4  TATTTTAAACCCAAAACAAATTTGAATATCAACTCCTCTAGGATCCAATATGGTTTTAAT 116
           *** ** **    *  ***** **  * *       ***  *  *******  * ***

LBCbarr2 GCCAACATCAATTTCGATGTTTCTGATGACTTTGGGAAATATGAAGGTAATAGACCGGGT 180
LBC-ori  GCTCGCATTGATATGCAGTATGAAGACGATAGTGGGACTAGAAAAGGCTCAAGACCCAAT 176
LBClus4  GCTCGCATTGATATGCAGTATGAAGACGATAGTGGGACTAGAAAGGGCTCAAGGCCCAAT 176
         **   ***  ** *  *   *   ** **   *****      * **    ** **   *

LBCbarr2 GCTTTTATTGGAAATGAAGTATCTCTTTATGGTAATTACAATTCTTATATCACGGATGAC 240
LBC-ori  GCATTTATGTCTAACACAGTTGCTTTTATAGGAAACTATGAAGGTATTATTGTTGATGAC 236
LBClus4  GCCTTTATGTCTAATACGGTTGCCTTTATTGGAAACTATGAAGGTGTCATCGTTGATGAC 236
         ** *****    **    **  *  **   ** ** **  *   *   **    ******

LBCbarr2 GTGCCAATCTATGATGGTATGCGGTCTGATGTGATCACTCCTGAAGGAGAGTTCAGCCAT 300
LBC-ori  ATTCCGATATTGGATGGTCTTAGGGCCGACATTTTTGATACTCATGGTGACTTAGACATG 296
LBClus4  ATCCCGATATTGGATGGCCTTAGAGCTGACATTTTTGATACTCATGGTGATTTAGACATG 296
          * ** ** *  *****  *  *  * **  *  *   * ** * ** ** **   *

LBCbarr2 GAATTAGCTGAGCAAGTGTTAAGTAAAGCGACATCGGACCGAGCATCCGCTCAGAAGATC 360
LBC-ori  GGCCTCGTTGAGGATGCATTGTCTAAGAGTACCATGATTAGAAGGAATGTACCAACTTAT 356
LBClus4  GGCCTCGTAGAGGATGCATTGTCCAAAAGTACTATGATTAGAAGGAATGTACCAACTTAT 356
         *   * *  *** * *  **    **    **   *    **      *  *  *

LBCbarr2 ACGCAATACTTGGCATACAATGGAGGTATGCGCAATTGTGTATCTATATTGTATAACTTA 420
LBC-ori  ACTGCTTACGCGAGTGAATTACTGTATAAGAGAAACCTTACATCTCTATTTTACAATATG 416
LBClus4  ACTGCTTACGCGAGTGAATTATTGTATAAGAGAAACTTAACATCTCTGTTTTACAATATG 416
         **    ***  *    *         ** * * **      **** * ** ** **  *

LBCbarr2 CTGCGTATGTATGTCATGAATGAGTTTGGCGAGGTGGTCTATGACCCAAACGAGATATTC 480
LBC-ori  CTCCGTTTATACTACATTAAAAAATGGGGCAGTATTAAGTATGAAAAAGATGCCATCTTT 476
LBClus4  CTCCGTTTATATTACATTAAAAAATGGGGTAGTATTAAGTATGAAAAAGATGCCATTTTT 476
         ** *** * **   *** **  * *  **     *    *****   * * *  ** **

LBCbarr2 TACGACAATGGACATGTGTGTATTAAAAATCATCAGATGTTTCCAAAACATCTAGAGAAG 540
LBC-ori  TATGATAATGGCCACGCCTGTCTTTTAAACAGGCAATTGTTTCCAAAGTCTCGTGATGCT 536
LBClus4  TATGATAATGGCCACGCCTGTCTTTTAAACAGGCAATTGTTTCCGAAGTCTCGTGATGCT 536
         ** ** ***** ** *  *** **  ***    **  ******* **   **  **

LBCbarr2 AAGTCATTAGCTAAGGCTGTT---GAGTACACTCCTGCTATGATCGAAGATGCTAGCACC 597
LBC-ori  TCTTTGGAATCAAGCCTCTCTTTGCCTGAGGCTGAAATTGCAATGCTTGATCCTGGCCTG 596
LBClus4  TCTTTGGAATCAAGCCTCTCTTTACCTGAGGCAGAAATTGCAATGCTTGATCCTGGATTG 596
            *    * * *       *       *  *      *   **    *** ** *

LBCbarr2 TTATGGCGCGAAGGTGACATACCTGAATGTCCTTCATTGATTTGGACAGGATTTATGCGT 657
LBC-ori  GAATTTCCAGAAGAGGATGTGCCTGC---------AATTTATGGCACGGCAGAGTGTCA 647
LBClus4  GAATTCCCAGAAGAGGATGTGCCTGC---------AATTTATGGCATGGCAGAGTGTCA 647
           **  *  ****  **  * ****          * *  * ***   **     **

LBCbarr2 GATAAGCAAGCTACTATCGTTTCACGGGCAGCAGGTGGATTCATTAACAGAGCACCTTTC 717
LBC-ori  TCCAGAGCAACGTGTATCTTAGGGCAAGCTTGCTCAGAGTTCGCGCCTCTGGCCCCCTTT 707
LBClus4  TCCAGAGCTACGTGTATCTTAGGGCAAGCCTGCTCTGAATTTGCGCCTCTGGCCCCCTTT 707
            *      *   **** *    *  **       *  **          ** ** **

LBCbarr2 GGAGTAGCTCATGAGTCAAAAAATCTTACTGAAAGATTAGGTATCATGGCAACTGGAGGA 777
LBC-ori  TCGATTGCGCATTATTCACCACAATTGACGAGAAAACTATTTGTCAATGCGCCCGCTGGG 767
LBClus4  TCAATTGCGCATTATTCACCACAATTGACAAGAAAACTATACGTCAATGCGCCCGCTGGG 767
             * ** *** * ***  * *  * **   ** * **    ***  **  * *  **
```

```
LBCbarr2 CTCTCTAATGTAGAAGATTATAACTATGGTCCTTCTGATATATTGTTCGTCCTTAACAAG 837
LBC-ori  ATTGAGCCTA---GCTCCGGGCGGTATACTCACGAGGATGTAAAAGATGCGATTACGATC 824
LBClus4  ATTGAGCCTA---GCTCCGGGCGGTATACTCACGAGGATGTAAAAAATGCGATTACGATC 824
          *      *               ***  **     *** **      *   ***  *

LBCbarr2 CTGGTGACAGATAACGGGCTTTACACTGACTTCACTTACGCTTATCTAATGTTGTTACAA 897
LBC-ori  CTTGTGTCTGCAAACCAGGCTTATACTGACTTTGAAGCAGCATACTTGATGCTTGCTCAA 884
LBClus4  CTTGTGTCTGCTAACCAGGCTTATACTGACTTTGAGGCAGCATACTTGATGCTTGCTCAA 884
         ** *** * *  ***  *  *** ********       ** **  * *** *    ***

LBCbarr2 GTATTAGTGTCTCCTATACCAAGGTCTGCTGAAGCTGCAGCATGGTTCGTCGGTGTCCAG 957
LBC-ori  ACGTTGGTCTCACCTGTACCACGCACTGCCGAAGCAAGTGCATGGTTCATCAATGCTGGC 944
LBClus4  ACATTGGTCTCACCTGTACCACGCACAGCCGAAGCTAGTGCATGGTTCATTAATGCTGGT 944
            ** ** ** *** ***** *  * ** *****    ********* *   **

LBCbarr2 ACAGTGTCTTTACCTAGGTTGGTGACTGACACAGGAAACATACCACAGTTATATCAGGGG 1017
LBC-ori  ATGGTCAATATGCCAACTTTGTCATGTGCAAATGGTTATTATCCAGCACTGACCAATGTC 1004
LBClus4  ATTGTTAACATGCCTATCTTGTCATGTGCTAATGGCTACTACCCAGCCTTAACAAATGTT 1004
         *   **    * ** *  ***     **  *  **  *    ***    *     * *

LBCbarr2 AAAGTCTATGCTAGGTTTAATGACTGGAAAGAGACTATGGCTAACTTCAGAGCATATCCT 1077
LBC-ori  AATCCTTACCACCGGCTAGACACATGGAAAGATACGTTAAATCATTGGGTGGCTTATCCC 1064
LBClus4  AATCCTTACCACCGGTTAGACACATGGAAAGACACTTTAAATCATTGGGTGTCTTACCCC 1064
         **    **     ** *  *    ******** **  *   * * *      * ** **

LBCbarr2 AAGTCATGTTTATTGCATAGTATTGTAGTGAATGAAGCTGTTTACGTTGAATTGAACAAT 1137
LBC-ori  GACATGCTGTTTTACCATTCAGTGGCAATGATTGAGAGCTGCTATGTTGAACTCGGGAAT 1124
LBClus4  GACATGTTATTTTACCATTCGGTGGCAATGGTTGAGAGCTGTTATGTTGAACTGGGGAAT 1124
          *       ** *  ***    * * * **  ***       ** ****** *    ***

LBCbarr2 CTTAACAGAGTGAGTCCTTTTGATGCAATTAATCGAGAAAATTTTGTTGAGGTAGCTGTA 1197
LBC-ori  GTGGCTCGTGTGTCAGACAGTGATGCAATAAACAAATACACTTTCACTGAGCTATCAGTG 1184
LBClus4  GTCGCACGTGTGTCAGACAGTGATGTAATTAACAAGTATACCTTCACTGAGCTATCTGTG 1184
          *     * ***        ***** *** **     * *  **   **** ** * **

LBCbarr2 CAAGGGCAGCCTGTCACAATGGGCGGGATATTGAATGATCTGACTTTAGTGTCGATGCGG 1257
LBC-ori  CAAGGACGGCCTGTTATGAATCGAGGAATTATTGTAGATCTGACACTTGTGGCAATGCGT 1244
LBClus4  CAAGGCCGGCCTGTTATGAACCGAGGGATTATTGTTGACTTGACACTTGTAGCAATGCGT 1244
         ***** * ****** *  *   * ** **  *    **  ****  * **  * *****

LBCbarr2 TATTCTAAGGAATTTGCACTGGCATATCCTGTGTCCACAGGACTTACAAGGACTAACATT 1317
LBC-ori  ACTGGTAGGGAGATCTCACTACCTTACCCGGTCAGCTGTGGCCTGACCCGTACAGACGCG 1304
LBClus4  ACTGGTAGAGAGATCTCACTACCTTACCCAGTTAGTTGTGGTTTGACCCGTACAGACGCA 1304
           *  **  **  *  ****  * ** ** **       **  * **  * **  **

LBCbarr2 ATGGCACAAGAAATAGCTCTTGAGGTACCAATAACAGTAAAAGACCTAGATGCACCTAAA 1377
LBC-ori  TTATTGCAAGGTACTGAGATTCACGTTCCAGTTGTTGTCAAAGATATCGACATGCCCCAG 1364
LBClus4  TTATTGCAAGGTACGGAGATTCATGTTCCAGTTGTCGTCAAAGACATGGACATGCCTCAG 1364
          *    ****  *  *   ** * ** *** *    ** *****  * **    **  *

LBCbarr2 TATTACAACATTGCAGAACCTATTATAACCGCAAATCAACATGCAACACTCATTGTTACT 1437
LBC-ori  TATTACAACGCGATTGATAAGGATGTTATTGAGGGGCAGGAAACTGTGATTAAAGTGAAA 1424
LBClus4  TATTATAATGCAATCGATAAGGATGTTATTGAAGGGCAAGAAACTGTAATAAAGGTGAAG 1424
         ***** **       **      * * *  *     **  *  *     * *  ** *

LBCbarr2 AATATGACGGCACCTTTGTATCCAGTAATGTCTTACGGGATTAATACTGATGACTATTAT 1497
LBC-ori  CAGCTGCCACCAGCTATGTATCCAATTTATACTTACGGGATCAACACTACTGAATTCTAT 1484
LBClus4  CAACTGCCACCAGCAATGTATCCGATTTATACTTATGGTATCAACACTACAGAGTTTTAC 1484
          *  ** *  ** *  *******  *     **** ** ** ** ***   ** *  **

LBCbarr2 TGTAACGATACGAACATCAGTATCAAATTTA---AGGCACCTGTGACTGCAAGTGGGATA 1554
LBC-ori  TCTGACCATTTTGAAGACCAGGTACAAGTTGAAATGGCACCAATCGATAATGGAAAAGCA 1544
LBClus4  TCCGACCATTTTGAGGACCAGGTACAAGTAGAAATGGCTCCTATCGATAATGGGAAGGCA 1544
         *   ** **    *   *    * **  *      *** **  *   *    *      *

LBCbarr2 TCTTTTACAGAAGCAGAAAATTTCTCAAAGTTTATGAACGTTATGCGTATGTGTGGGTAT 1614
LBC-ori  GTTTTTAACGATGCAAGAAAGTTTCGAAATTTATGTCCATAATGCGCATGATGGGGAAT 1604
LBClus4  GTCTTTAATGATGCTCGAAAATTCTCTAAATTCATGTCTATAATGCGCATGATGGGGAAT 1604
            ****  ** **   *** ** ** ** ** ***    * ***** ***   *** **

LBCbarr2 AACGTTGAGGCCAGAGAGATGTTTTCAGGCAAGACTGTCATTAACTGGGCAGATAATGCT 1674
LBC-ori  GATGTTACTGCTACTGATTTAGTTACAGGTAGAAAAGTGTCGAATTGGGCCGACAACTCA 1664
LBClus4  GATGTTACGGCAACTGATTTAGTAACAGGTAGAAAGGTGTCGAATTGGGCCGACAACTCA 1664
```

```
          * ***   ** *  **  *  *  **** *  *  **    ** ***** ** **  *

LBCbarr2 AGTGGAAGATTTTTATATACAGATGCTCAAGTTGGTGAGGATATACATTATCATGTACCT 1734
LBC-ori  TCAGGGCGTTTCTTGTACACGGATGTGAAGTATGAAGGACAAACTGCCTTTTTGGTTGAT 1724
LBClus4  TCAGGGCGTTTCTTATACTCAGATGTCAAGTATGAAGGCCAAACTGCTTTTTTGGTTGAT 1724
            **  * ** ** **  * ****   *   **  *   * *     * *   **   *

LBCbarr2 TACGCCAACATTCATAGGCGGAGTAATTCATGGTTAAACGTGATAACCTTTAGAGGTGAA 1794
LBC-ori  ATGGATACTGTCAAGGCGAGAGACCACTGTTGGGTGTCAATTGTTGATCCTAATGGTACA 1784
LBClus4  ATGGACACTATTAAGGCTAGAGACCATTGCTGGGTGTCAATTGTGGATCCTAATGGCACG 1784
                *  *   *  *     *     * *  *** *     *  *      **  **

LBCbarr2 ACAGCCTTCAATGCGAACATCACAGCTGTGAATTTTAAAATGTTCAACGGCCCATCTGAA 1854
LBC-ori  ATGAACTTGTCATATAAGATGACCAATTTTAGAGCAGCAATGTTTTCTAGAAACAAGCCC 1844
LBClus4  ATGAGCTTGTCATATAAGATGACAAATTTCAGGGCAGCAATGTTTTCAAGAAACAAACCT 1844
         *    ***       ** ** **   * * *       ******     *

LBCbarr2 GTACTAATGACAGGGCCTCGAGTTATGCCAGTACAGATGAATGACTTCGTAGACAAGGCA 1914
LBC-ori  TTGTATATGACAGGGGGGTCAGTCAGGACCATAGCTACTGGCAATTATCGAGATGCTGCT 1904
LBClus4  CTATACATGACAGGTGGATCAGTCAGGACTATAGCTACTGGTAACTACCGTGATGCCGCT 1904
          *    ********      *** * * *  **   *      * *     **    **

LBCbarr2 ATTGAAATTGCTTCTCGTACTGACACGATTAAAATCTTACCTCTACGGAAAACTATGCTG 1974
LBC-ori  GAAAGATTACGTGCAATGGATGAAACGCTCAGATTAAAACCTTTTAAGATTACTGAGAAG 1964
LBClus4  GAGAGGTTGCGTGCAATGGACGAAACGTTGAGGTTAAAGCCTTTTAAGATTACTGAGAAG 1964
                *   * *       ** *** * *   *    *** *   **  ***  *  *
           ∩
LBCbarr2 CCGGATTTTCGATTCCTCAGGTATCAGTTCCCGGGTGTGTCGGGGATGGGGAATACTGTC 2034
LBC-ori  TTGGATTTTCGTGTAGCAGCTTACGCGATACCAAGTTTGTCGGGCAGCAATATGCCATCC 2024
LBClus4  TTGGATTTTCGTGTAGCAGCTTATGCGATACCAAGTCTGTCGGGCAGCAATATGCCATCC 2024
         *********  *       **    * * **   ** ******* *     *   *   *
```

-1 Frame-shift site *Stem-loop for frame-shift (ΔG=-21.9 kJ/mol in LBCbarr2, ΔG=-35.1 kJ/mol in LBC-ori)*

```
LBCbarr2 TTGCCACGGGAGAGCACACAACAGTTTGGCCAGGAAGAACAAGAGCTAGTGAATCCAGAG 2094
LBC-ori  TTACACCATCAGGAACAACTACAGATATCAGAAGTGGACGCGGAACCAATCAATCCTATA 2084
LBClus4  TCACACCATCAGGAACAACTACAGATATCAGAAGCGGACGCGGAGCCGATCAATCCTGTG 2084
         *  *  *   **     ** **** *     * *  **    ** *   * *****

LBCbarr2 GCGAGAGATATGTCAATCCAGGATGTAGAGTAG-------------TACCGACATATCTA 2141
LBC-ori  GGAGAGGACGAACTTCCACCGGATATAGAATAGGTGTCGAAGACGATGAGGACTTAGATA 2144
LBClus4  GGAGAGGACGAACTTCCACCGGATGTAGAGTAGGTGTTGAAGATGATGAAGACTTAGATA 2144
         *     **          * **** **** ***             *   *** **  **
```

Stop of Gap LBC and LBCbarr

```
LBCbarr2 A----------CAATAGAC-------------------GGG---GAGATGACGACAACGC 2169
LBC-ori  TTGGTACGGTCAAATACATTGTGCCATTGTATTTGAACGGTGATAATGTGGCACAAAATT 2204
LBClus4  TTGGTACGGTCAAATACATTGTGCCATTGTATTTGAACGGTGATAATGTGGCACAAAACT 2204
                     **** *                    **     *  ** *   **

LBCbarr2 CAGAGGAAGCTGACAAAGTGCT---TCTGGCCATGTATTATTCAAACCAGAAAGTGATTA 2226
LBC-ori  GTTTAGAAGCAACACACGTGCTTATCAAAGCTTGTAGTATTGCGAACCGGATTGTAGATG 2264
LBClus4  GTTTAGAAGCAACACATGTACTTATTAAAGCTTGTAGTGTTGCAAACCGGATTGTAGATG 2264
              *****     * ** **       **      *  * * **** **  **   *

LBCbarr2 AGACTAAGGCAGGATCTTTCACTCAGACTGGATTATCTAAGCAGATGATAGTTCATAATG 2286
LBC-ori  ACGGAGAGGGTCACTGTTTCACACAGCAAGGGCTGGCGCAGCAGTGGATCTTCCATAGGG 2324
LBClus4  ATGGAGAGGGTCACTGCTTTACACAGCAAGGGCTAGCACAGCAGTGGATCTTCCATAGGG 2324
         *     ***     *  ** ** ***   **  *  *  *****  ***  * ****  *

LBCbarr2 GGATGACAGTTAATACCGACGTCATCAGAATTGGGGATGTCAATTGTTATTATGTACGGT 2346
LBC-ori  GGGAGATGATATTTGTGAAGGCGGTACGCATTGGTCAACTCAATGCATATTATGTAGACT 2384
LBClus4  GGGAGATGATATTTGTGAAGGCAGTACGCATTGGCCAGCTCAATGCATACTATGTAGACT 2384
         **  **   *   *    * *   *  * *****  *  *****   ** ******   *

LBCbarr2 TGAAGTTTTGTGATGCAGCATCAAAAGAAATTACACGTCAAGCAGGAGCAACGATGTGTG 2406
LBC-ori  ATAAGAACGTCACAAATTATAGTCTTAAAACCGCTGCTCAAGTAGGAGCGACGATATCAA 2444
LBClus4  ATAAGAACGTCACGAATTACAGTCTTAAAACTGCTGCTCAAGTGGGAGCGACGATATCAA 2444
           ***                      ***   *   *****  ***** ***** *

LBCbarr2 GTAACTTCAGACACTTGTTTGCGAACTCAACTGGTACAGTATACGATTGGCTGGACCGGG 2466
LBC-ori  ATAACTTACGCCACGGATTTGTTGACAATCAACAAGACGCATACACGCGCTTGGTTGCCA 2504
LBClus4  ACAACTTACGTCATGGATTCGTTGACAACCAGCAAGACGCATACACGCGCTTGGTTGCCA 2504
           *****  * **    ** *   **            * ****    *  ***

LBCbarr2 ACAAAACCGTATTAAGAATGTACGACAGGCTTGCTAGTTACAAGTATGACTACAATATAG 2526
```

```
LBC-ori  ACTACTCTG---ATACGCGGAAGTGGATACGTGACAATTTTACATATAATTATAAT---A 2558
LBClus4  ATTACTATG---ATACACGGAAGTGGATACGTGACAATTTTACATATAATTATAAT---A 2558
         *  *    *     *    * *    *  * **  * **  *  *** * ** ***

LBCbarr2 CTGATAAAATAACAGAAAAAATAAGTCAGTTTCATCATGTACACGTGAGGCTGAAGGACT 2586
LBC-ori  TGGAGAAAGAAAAGTATAGGATAACCCAATACCACCATACACATGTGAGGTTGAAAGATT 2618
LBClus4  TGGAGAAAGAAAAATATAAGATAACTCAATATCACCACACACACGTTAGATTGAAAGATT 2618
           ** ***  **   * *  ****  ** *  ** **   *** ** **  **** ** *

LBCbarr2 TATATGCTAATAAGGATATTGTGACAGTAAACGGTCATGAAGCATATCTCGTGAACTTAC 2646
LBC-ori  TGTTTCCATCCAGGAAAATAGTTAAACTAGAGGGATATGAAGCCTTGTTGGCAATGATGC 2678
LBClus4  TGTTTCCATCTAGGAAAGTAGTCAAACTAACAGGATACGAGGCCCTACTGGCAATGATGT 2678
         * * * *    * * *  * ** * * **   **  * ** **     * *  *   *

LBCbarr2 TTGATAAGTACACAGACAAAGCTATTACACATGCTACTTTCATAACCTATCTAAGAGCAC 2706
LBC-ori  TAGACAGGTTTAACAACATAGAGTCAACACATGTAACTTTCTTCACATATTTAAGAGCAC 2738
LBClus4  TAGATAGGTTCAACAATATAGAGTCAACACATGTCACCTTTTTACATACTTGAGGGCAT 2738
         * ** * **  *   * * **     *******  ** **  * ** **  * ** ***
.
LBCbarr2 TGCCTGTTAGGGAAGCAAGGCTCTTCATAAAATTAGTCATTAAGTATGGTAGCATAGACC 2766
LBC-ori  TACCTGACCGTGAAAAAGAAGTCTTTATTAGCTTAGTCTTAAACTATAATGGCCTTGGCA 2798
LBClus4  TGCCTGATCAAGAGAGGGAAATCTTCATTAGCTTAATTTTGAACTACAACGGGCTTGGTA 2798
         * ****     **        **** ** *  *** *  * ** **     *  * *

LBCbarr2 AAGAATGGTTAAAAGAAGAAGGGACTGTAGCAAAGCAAATACAGGGCAGCACTGACTTTG 2826
LBC-ori  GAGAGTGGTTGAAGTCTGAAGGTGTTAGGGCTAAACAAGCACAAGGTACTGTGAAATACG 2858
LBClus4  GAGAGTGGTTAAAGTCAGAAGGTGTTAGGGCAAAGCAAGCGCAAGGTACTGTAAAGTATG 2858
          *** ***** **    *****   *   ** ** ***   ** ** *      * *  *

LBCbarr2 ATCTAAGCAAAATTTTTGAGCTTAATGTACTGCATAATCGGGTTGAAACTACAGTTGATT 2886
LBC-ori  ATATGAGTAAACTATTTGAACTGAATGTACTAGAGAACGGAGTTGACGAAGAAGTTGACT 2918
LBClus4  ATATGAGTAAATTATTTGAGCTCAATGTGTTGGAAAATAGGGTTGACGAAGAGGTTGACT 2918
         ** * ** *** * ***** ** *****  *  * **  * *****       ***** *

LBCbarr2 GGAATGAAGAAAGAGAACATAGGACGCATCCTGATACAGTGAATATATCTTATCCAGATG 2946
LBC-ori  GGGAGAAAGAGAAACGCAACAGGTCAGATATCAAGACTGTTAACATAAGTTATGCAAAAG 2978
LBClus4  GGGAAAAAGAAAAACGGAACAGGACGAACATCAAGACTGTAGACATAAGTTATGCAAAAG 2978
         ** *  **** * *    * *** *  *     * ** **  * ***  **** ** * *

LBCbarr2 TCTATACTAAGTGCAGAGAATTGTTCGAATTAGCAAAGCAGGAAGGAAAGTCACCTATTA 3006
LBC-ori  TTCTCGAACATTGTAGAGAGCTATTCATCATGGCGAGGGCCGAAGGGAAACGGCCAATGA 3038
LBClus4  TTCTTGAGCACTGTAGAGAACTATTTATCATGGCAAGGGCTGAAGGAAAGCGGCCAATGA 3038
         *        * ** *****  * **     * ** * *   ***** **    ** ** *

LBCbarr2 AGATGGACTGGAATAAGTACTGGATGCAGCGAGCCTCAATAATGCCATCTGGTGCAGTCC 3066
LBC-ori  GGATGAAATGGCAAGAGTACTGGAGGCAGAGAGCAGTTATCATGCCAGGTGGATCGGTCC 3098
LBClus4  GGATGAAATGGCAGGAGTATTGGAGGCAGAGAGCAGTTGTGATGCCAGGCGGTTCAGTCC 3098
          **** * *** *  **** **** **** ****     * ******   **  * ****

LBCbarr2 ACTCACAACATAGTGAGCTTGACAAGTATGTGAGGAAATTACCTCGTGAAGTCAAAAACA 3126
LBC-ori  ACAGTCAACATCCAGTCGAACAGGACGTGATTAGAGTATTACCCAGAGAAATCAGAAGTA 3158
LBClus4  ACAGTCAGCATCCAGCCGAACAAGATGTGATTAAGATATTACCCAGGGAAATTAAGAGTA 3158
         **   ** ***   *      *  *     * *    ******  * *** * *  *  *

LBCbarr2 AGAAGGGGTTGGCTGCCTCGCTACCTAATATAGACCAGAGGTACTTTCTGAGTAAAATAC 3186
LBC-ori  AGAAGGGGGTGGCAAGTGTCATGCCATACAAAGAACAGAAGTATTTCACGTCCAGAAGGC 3218
LBClus4  AGAAGGGGTTGGCAAGTGTTATGTCATATAAAGAACAAAAATATTTCACATCAAGGCGAC 3218
         ******** ****        *  *  * * *** ** *  ** **       *     *

LBCbarr2 CTTCTATTGAAGCGTATGTCAGTACAAAGTATGAATGGGGAAAAGTCAGGGCTTTATATG 3246
LBC-ori  CGGAAATACACGCTTACACTTCAACGAAATACGAGTGGGGAAAAGTGAGGGCACTATATG 3278
LBClus4  CAGAAATACATGCTTACACTTCCACAAAATATGAGTGGGGAAAAGTGAGAGCGTTGTACG 3278
         *    **  *  ** **       ** ** ** ** *********** ** **  * ** *
```

Highly conserved RdRp domain

```
LBCbarr2 GATGTGATTTCAGCAGTCATGTTAATGCAGACTTTGGATTATTAAATTGTGAAGATACCT 3306
LBC-ori  GGTGTGATTTTTCATCACATACAATGGCTGATTTTGGATTGTTACAATGCGAGGATACAT 3338
LBClus4  GGTGTGACTTTTCATCACACACGATGGCTGATTTTGGGCTGTTACAGTGTGAGGATACAT 3338
         * ***** **       **    *  ** ** *****  * *** * ** ** ***** *

LBCbarr2 TCCCTCACTTTATACCTACTGGGCCACAAGCTACTGAGGGGTACGTAAAAACACTTTTAA 3366
LBC-ori  TCCCGGGCTTTGTACCAACAGGGTCTTACGCCAATGAGGATTATGTCAGGACCAGAATTG 3398
LBClus4  TCCCCGGATTTGTACCAACGGGGTCATATGCTAACGAGGACTATGTCAGGACTAGGATAG 3398
         ****    *** **** ** *** *  * ** *  ****  ** ** *  **     *
```

```
LBCbarr2 AAACAGCTAAATATAATGTGCCTTTCTGTTATGACTATGATGACTTCAACAGTCAGCATT 3426
LBC-ori  CTGGGACTCACTCATTGATCCCTTTCTGTTACGATTTCGATGATTTCAACAGCCAACATT 3458
LBClus4  CAGGGACACACTCGTTGATTCCTTTCTGTTATGACTTTGATGATTTTAATAGTCAACACT 3458
               *  * *      * *********** ** *  ***** ** ** ** ** ** *

LBCbarr2 CAAAGTCAAGTATGCAAGCAGTGATAGACGCTTGGATAAATACATATTTCGATGACCTGA 3486
LBC-ori  CAAAGGAAGCCATGCAAGCAGTGATTGATGCATGGATATCTGTCTATCACGATAAGTTAA 3518
LBClus4  CAAAGGAAGCTATGCAAGCAGTTATCGATGCATGGATATCTGTTTACCACGATAAGTTAA 3518
         *****  *   *********** ** ** ** ******  *   **   **** *  * *

LBCbarr2 CTGAAGACCAGATTGCCTCAGCCCTATGGACTAGAGAAAGTGTTGGTGAAATGTTTGTTA 3546
LBC-ori  CAGATGACCAGATAGAGGCGGCAAAGTGGACACGAAACTCGGTAGATAGAATGGTCGCTC 3578
LBClus4  CAGATGATCAAGTAGAAGCAGCAAAGTGGACAAGAAACTCAGTAGATAGAATGATCGCAC 3578
         * ** ** **  * *   * **    *****  ** *    ** * *  **** * *

LBCbarr2 ATGATGTAAAGAACAGTGTCAGATATGAAGCCATGGGAACTCTGTTTAGCGGATGGAGGC 3606
LBC-ori  ACCAACCTAACACTGGTGAGACTTATGATGTTAAAGGGACACTGTTTAGTGGCTGGCGAT 3638
LBClus4  ACCAAACGAACACCGGCGAGACTTATGATGTCAAAGGGACACTATTTAGTGGCTGGCGAT 3638
         *  *    ** *   * *  *  ***** *  *  ** ** ** ***** ** *** *

LBCbarr2 TGACAACTTTTATAAACACCGCTCTTAATTATGCATATCTTGCTAAGGCAGGGATAAATA 3666
LBC-ori  TAACAACATTTTTCAATACGGCGTTGAACTATTGCTACCTGGCTAATGCAGGTATAAACT 3698
LBClus4  TAACTACATTTATCAACACAGCATTAAACTATTGCTACTTGGCCAATGCAGGTATAAACT 3698
         * ** ** *** * ** ** **  * ** ***   **  * ** ** ***** *****

LBCbarr2 AATTAACCAATATAAGTATCCATAATGGTGATGATGTCTATGCAGGGACGAGAAATTTGA 3726
LBC-ori  CACTAGTGCCAACGAGTCTCCATAATGGTGATGATGTTTTTGCAGGGATAAGGACAATAG 3758
LBClus4  CACTAGTACCAACGAGCCTCCACAACGGTGATGATGTCTTTGCAGGTATAAGGACGATAG 3758
          * **      *  **  **** ** *********** * ****** *  ** *   *

LBCbarr2 AAGATATTGCTAGGCTTTTGAAGAATAGCAAGCAGATGGGAATACGTGCCAATACCACGA 3786
LBC-ori  CTGACGGTATTTCTTTGATCAAAAACGCCGCAGCCACGGGAGTTCGCGCTAATACAACTA 3818
LBClus4  CTGACGGTATTTCATTAATCAGAAATGCGGCAGCTACTGGAGTACGTGCAAATACGATTA 3818
           **   *  *    *  * *  **          *  *** * ** ** ***** *  *

LBCbarr2 AAATGAGCATTGGTACTATCGCTGAGTTTTTACGTGTCGACATGAGGGCTGAAAAACCTA 3846
LBC-ori  AAATGAACATTGGTACGATAGCAGAGTTTTTGAGAGTTGATATGCGTGCAAAAAATAGTA 3878
LBClus4  AGATGAACATAGGAACAATAGCGGAATTTTTGAGAGTTGATATGCGTGCAAAAGACAGTA 3878
         * **** *** ** ** ** ** ** *****  * ** ** *** * **  ** *   **

LBCbarr2 CTAGTGCTCAGTACTTAACTAGAGGGATATCTACTTTCGTACATGGTAGGATAGAAAGTG 3906
LBC-ori  CTGGCAGTCAGTATTTAACAAGAGGGATTGCTACCTTCACGCACAGTAGGGTTGAGTCTG 3938
LBClus4  CTGGTAGCCAATATTTGACAAGAGGGGTTGCTACTTTTACACACAGTAGGGTCGAGTCAG 3938
         ** *    ** ** ** ** ****** *  **** **    **  ***** * **    *

LBCbarr2 AGGCTCCAGTCGGGTATAGGGCTATGGTTAGTGCTTATAAGACGCGATACGATGAAGTGG 3966
LBC-ori  ATGCACCACTGACATTGCGCAATCTAGTATCTGCTTACAAAACCAGATATGACGAGATTT 3998
LBClus4  ATGCCCCACTAACATTACGTAATCTAGTATCTGCATACAAGACCAGGTATGATGAGATCA 3998
         * ** *** *    *   *   * * **   *** ** ** **  * ** ** **  *

LBCbarr2 TAGAAAGAGGAGGAAATGCGCAACGACTAAGGCATTTGTATCGTAAACAACTATTTTTCG 4026
LBC-ori  TAGCTCGTGGCGCAAGCATCGATAACATGAAGCCACTCTATCGTAAGCAATTATTTTTTG 4058
LBClus4  TAGCTCGTGGTGCTAACGTTGATGATATGAAACCACTTTATCGGAAACAATTATTTTTCG 4058
         ***   * ** *  *      *     * *  *   * ***** ** *** ******* *

LBCbarr2 CTCGTAGAAAGTTTGATGTGTCTGAAGAAATGCAAGAAAAATTGTTAAAGACGCACGTGC 4086
LBC-ori  CTAGAAAGTTGTTCAATGTCGAGAAGGACATTGTTGACAATCTGATAACGATGGACATAT 4118
LBClus4  CTCGCAAGTTATTCAATGTCGAGAAGGGTATAATTGATAATTTGATAACGATGGACATAT 4118
         ** * *     **  ****     * *  **    ** **  ** *** ** * ** *

LBCbarr2 AAGCTGGCGGTCTATCAGTCAATGGTAAGATAGGAAACTACACATTGGAAGATGACAGCC 4146
LBC-ori  CATGTGGCGGTTTGCAAGAAAAGGGTAGGGTATCAGAGATGGTGTTACAGGAGGTTGACA 4178
LBClus4  CATGTGGTGGTTTGCAGGATAAAGGTAAGGTGTCAGAAGTGGTGTTACAGGAAGTTGATA 4178
          *  *** *** *    *  ** **** * *   * *       **  * ** *

LBCbarr2 TTGCATACGCAGATATTGACGTGAATGAAATAAGC------AGCTTATTACAGCCTGGAG 4200
LBC-ori  TTGAGAATATAGATAGTTATAGGAAGACAAGGATGATCGCCAAACTGATTGACAAGGGGG 4238
LBClus4  TTGAAAATATAGACAGTTATAAGAAGACTAGGATGATCGCAAAACTGATCGACAAGGGGG 4238
         ***   *   *** * * *   ***    *  *        *   *  *  *    ** *
```

Stem-loop for packaging ΔG=-7.1 kJ/mol

```
LBCbarr2 TTAAGGACTACGTGACTAGTTTGAAGTCTTTGTATCCGGAAATACGGGAATTTATAACCG 4260
LBC-ori  TTGGCGATTATACTGCATTCCTGAAAACTAACTTTTCCGAGATAGCTGATGCTATCACAA 4298
```

```
LBClus4   TTGGTGATTACACAGCATTCTTAAAAACTAACTTCTCCGAAATAGCTGATGCTATCACAA 4298
          **   ** **     *     * **  **   *   * ** ***   **   *** **

LBCbarr2  AAAAGACAGTGAGAAGGACACTTATGAAAGCCTTCAACATAAATAAAAACACGATATGTA 4320
LBC-ori   GAGAGACACGTGTAGAGTCAGTGACCAAGGCTTATAATGTTAAGAAGAAAACGGTCGTAC 4358
LBClus4   AGAAACATGTTGTAGAGTCATTGACCAAGGCCTACAATGTCAAGAAGAAAACGGTCAGAA 4358
             *         *  * ** * *  ** ** *  **  * ** ** ** *** *

LBCbarr2  TCAAACCAGCCAGTAGCAAGGCGATATATAATCAGATAGCACTAAGAGGTGCATGGAATG 4380
LBC-ori   GCGCGTTTAGGGACCTAAGCGCAGCATATCATGAAAGAGCGGTGAGACATGCTTGGAAGG 4418
LBClus4   GCGCGTTTAGGGACCTAAGCGCAGCATATCATGAGAGAGCAGTTAGACATGCTTGGAAGA 4418
           *               *  **   **** ** * * ***  * ***  *** *****

         Stem-loop for packaging (ΔG=-52.3 kJ/mol)
LBCbarr2  ATGACTCTAATTTCAGAATATTTAACAGAGTTAGACAGGGTGTGAGTAACGTGATAGGCG 4440
LBC-ori   GGATGAGTGGACTACACATAGTCAACAGGATTCGTATGGGAGTGAGCAACTTAGTAATGG 4478
LBClus4   GGATGAGCGGACTACATATAGTTAACAGGGTTCGTATGGGCGTAAGCAACTTAGTAATGG 4478
                      *    *** * *****  ** *   *** ** ** *** *  **   *

LBCbarr2  TATTAGGTAAACTGTCCAATGCGCATGCAAAAGTATTAAGCGAAACAAATGACCCTATCA 4500
LBC-ori   TTGTTAGCAAAATCAATCCTGCAAAAGCTAATGTGCTAGCCAAATCAGGAGATCCTACAA 4538
LBClus4   TTGTTAGCAAAATCAATCCTGCGAAAGCTAATGTGTTAGCCAAATCAGGGGATCCCACCA 4538
          *  *  * *** *      ***  * ** ** **  **  * ** **   ** ** *  *

                                                  Stop of LBCbarr2 Gag-Pol
LBCbarr2  AATGGTTGTCTATATATATAACATAGATGTAATACCAAGAAAGTTGTGACCTATATGACA 4560
LBC-ori   AATGGCTTGCAGTCCTTACATGATATACAGGCAACCACATAAGACCTGAGAACAAAGAGT 4598
LBClus4   AATGGCTTGCTGTCCTTACATGATATACAGGCAACAACATAAGACCTGAGAACAAAGAGT 4598
          ***** *  *  *   ** *  *** *      ** *   ***   ***     * **

                Stop of LBC-ori and LBClus4 Gag-Pol     Stem-loop for replication (ΔG=-13.8 kJ/mol)
LBCbarr2  ACGGC------------    4565
LBC-ori   ACATACGATACTACGCA    4615
LBClus4   ACATACGATACTACGCC    4615
          **
```

Stem-loops for replication in LBV-ori (ΔG=-7.5 kJ/mol and ΔG=-2.9 kJ/mol, respectively)

Figure S1. Multiple sequence alignment between ScV-LBC1-original, ScV-LBClus4, and TdV-LBCbarr2 (+) strand nucleotide sequences (cDNA). 5´GAA(A/T)TT conserved motif (5´ conserved), translation initiation (start of Gag and Gag-Pol, or internal possible start ATG in Pol ORF of LBC1-original, LBClus4 and LBCbarr2), termination codons (stop of Gag and stop of Gag-Pol), ribosome frameshifting site (-1 frameshift site), frameshifting associated sequence (stem loop for frameshift), packaging signal (stem loop for packaging), and replication signal (stem loop for replication) are indicated, shaded and/or underlined in the nucleotide sequence. The highly conserved RdRp domain located in the central third of Pol is also underlined. ⋂, ribosomal frameshift. Asterisks (*) indicates identical nucleotide positions.

```
LBCbarr2 MFSTLNNLLPDTLNPRKNFKLTHHRIKYGMNANINFDVSDDFGKYEGNRPGAFIGNEVSL 60
LBC-ori  MSSLLNSLLPEYFKPKTNLNINSSRVQYGFNARIDMQYEDDSGTRKGSRPNAFMSNTVAF 60
LBClus4  MSSLLNSLLPEYFKPKTNLNINSSRIQYGFNARIDMQYEDDSGTRKGSRPNAFMSNTVAF 60
         * * **.***: ::*:.*:::.  *::**:**.*::: .** *. :*.**.**:.* *::

LBCbarr2 YGNYNSYITDDVPIYDGMRSDVITPEGEFSHELAEQVLSKATSDRASAQKITQYLAYNGG 120
lbc-ori  IGNYEGIIVDDIPILDGLRADIFDTHGDLDMGLVEDALSKSTMIRRNVPTYTAYASELLY 120
lbclus4  IGNYEGVIVDDIPILDGLRADIFDTHGDLDMGLVEDALSKSTMIRRNVPTYTAYASELLY 120
          ***:. *.**:** **:*:*::  .*::.  *.*:.***:*  *  ..  . * * :
                                                   His156 cap-snatching
LBCbarr2 MRNCVSILYNLLRMYVMNEFGEVVYDPNEIFYDNGHVCIKNHQMFPKHLEKKSLAKAVEY 180
LBC-ori  KRNLTSLFYNMLRLYYIKKWGSIKYEKDAIFYDNGHACLLNRQLFPKSRDASL-ESSLSL 179
LBClus4  KRNLTSLFYNMLRLYYIKKWGSIKYEKDAIFYDNGHACLLNRQLFPKSRDASL-ESSLSL 179
          ** .*::**:**:* ::::*.: *: : *******.*: *:*:***  : .   .::.

LBCbarr2 T---PAMIEDASTLWREGDIPECPSLIWTGFMRDKQATIVSRAAGGFINRAPFGVAHESK 237
LBC-ori  PEAEIAML-DPGLEFP---EEDVPAILWHGRVSSRATCILGQACSEFAPLAPFSIAHYSP 235
LBClus4  PEAEIAML-DPGLEFP---EEDVPAILWHGRVSSRATCILGQACSEFAPLAPFSIAHYSP 235
             **: * .  :      : *:::* * : .: : *:.:*.. *   ***.:** *

LBCbarr2 NLTERLGIMATGGLSNVEDYNYGPSDILFVLNKLVTDNGLYTDFTYAYLMLLQVLVSPIP 297
LBC-ori  QLTRKLFVNAPAGIEPSS-GRYTHEDVKDAITILVSANQAYTDFEAAYLMLAQTLVSPVP 294
LBClus4  QLTRKLYVNAPAGIEPSS-GRYTHEDVKNAITILVSANQAYTDFEAAYLMLAQTLVSPVP 294
         :**.:* : * .*:.  .  .*  .*:  .:. **: *  ****  ***** *.****:*

LBCbarr2 RSAEAAAWFVGVQTVSLPRLVTDTGNIPQLYQGKVYARFNDWKETMANFRAYPKSCLLHS 357
LBC-ori  RTAEASAWFINAGMVNMPTLSCANGYYPALTNVNPYHRLDTWKDTLNHWVAYPDMLFYHS 354
LBClus4  RTAEASAWFINAGIVNMPILSCANGYYPALTNVNPYHRLDTWKDTLNHWVSYPDMLFYHS 354
         *:***:***:..  *.:* *   .*  * * : : * *:: **:*: :: :**.  : **

LBCbarr2 IVVNEAVYVELNNLNRVSPFDAINRENFVEVAVQGQPVTMGGILNDLTLVSMRYSKEFAL 417
LBC-ori  VAMIESCYVELGNVARVSDSDAINKYTFTELSVQGRPVMNRGIIVDLTLVAMRTGREISL 414
LBClus4  VAMVESCYVELGNVARVSDSDVINKYTFTELSVQGRPVMNRGIIVDLTLVAMRTGREISL 414
         :.: *: ****.*: ***  *.**: .*.*::***:**   **: *****:** .:*::*

LBCbarr2 AYPVSTGLTRTNIMAQEIALEVPITVKDLDAPKYYNIAEPIITANQHATLIVTNMTAPLY 477
LBC-ori  PYPVSCGLTRTDALLQGTEIHVPVVVKDIDMPQYYNAIDKDVIEGQETVIKVKQLPPAMY 474
LBClus4  PYPVSCGLTRTDALLQGTEIHVPVVVKDMDMPQYYNAIDKDVIEGQETVIKVKQLPPAMY 474
          **** *****: : *   :.**:.***:* *:***  :  :  .*.:.: *.::   :*

LBCbarr2 PVMSYGINTDDYYCNDTNISIKF-KAPVTASGISFTEAENFSKFMNVMRMCGYNVEAREM 536
LBC-ori  PIYTYGINTTEFYSDHFEDQVQVEMAPIDNGKAVFNDARKFSKFMSIMRMMGNDVTATDL 534
LBClus4  PIYTYGINTTEFYSDHFEDQVQVEMAPIDNGKAVFNDARKFSKFMSIMRMMGNDVTATDL 534
         *: :***** ::*.:. : .::.   **:   .   *.:*.:*****.:*** * :* * ::

LBCbarr2 FSGKTVINWADNASGRFLYTDAQVGEDIHYHVPYANIHRRSNSWLNVITFRGETAFNANI 596
LBC-ori  VTGRKVSNWADNSSGRFLYTDVKYEGQTAFLVDMDTVKARDHCWVSIVDPNGTMNLSYKM 594
LBClus4  VTGRKVSNWADNSSGRFLYSDVKYEGQTAFLVDMDTIKARDHCWVSIVDPNGTMSLSYKM 594
         .:*:.* *****:******:*.:    :  : *   .:: *.:.*:.::  .*   :. ::
                                                   Gag domain ◄► Pol domain
LBCbarr2 TAVNFKMFNGPSEVLMTGPRVMPVQMNDFVDKAIEIASRTDTIKILPLRKTMLPGFSIPQ 656
LBC-ori  TNFRAAMFSRNKPLYMTGGSVRTIATGNYRDAAERLRAMDETLRLKPFKITEKLGFSCSS 654
LBClus4  TNFRAAMFSRNKPLYMTGGSVRTIATGNYRDAAERLRAMDETLRLKPFKITEKLGFSCSS 654
         * ..  **.  . : ***  *  :  .:: * * .: :  :*::: *:: *   ***  .

LBCbarr2 VSVPGCVGDGEYCLAT-GEHTTVWPGRTRASESR-G-------------ERYVNPGCRV 700
LBC-ori  LRDTKFVGQQYAILTPSGTTTDIRSGRGTNQSYRRGRTSTGYRIGVEDDEDLDIGTVKYI 714
LBClus4  LCDTKSVGQQYAILTPSGTTTDIRSGRGADQSCGRGRTSTGCRVGVEDDEDLDIGTVKYI 714
         :     **:    *:  *  * :  **    ..   *                :  :.    :

LBCbarr2 IPTYLTIDGEMTTTPEEADKVLLAMYYSNQKVIKTKAGSFTQTGLSKQMIVHNGMTVNTD 760
LBC-ori  VPLYLNGDNVAQNCLEATHVLIKACSIANRIVDDGEGHCFTQQGLAQQWIFHRGEMIFVK 774
LBClus4  VPLYLNGDNVAQNCLEATHVLIKACSVANRIVDDGEGHCFTQQGLAQQWIFHRGEMIFVK 774
         :* **. *.    .  * :. :: *    :*: * . :. .*** **::* *.*.*  : ..

LBCbarr2 VIRIGDVNCYYVRLKFCDAASKEITRQAGATMCGNFRHLFANSTGTVYDWLDRDKTVLRM 820
LBC-ori  AVRIGQLNAYYVDYKNVTNYSLKTAAQVGATISNNLRHGFVDNQQDAYTRLVANYSDTRK 834
LBClus4  AVRIGQLNAYYVDYKNVTNYSLKTAAQVGATISNNLRHGFVDNQQDAYTRLVANYYDTRK 834
         .:***::*.***  *     * : : *.***:..*:** *.:.    .*  *  :    *

LBCbarr2 YDRLASYKYDYNIADKITEKISQFHHVHVRLKDLYANKDIVTVNGHEAYLVNLLDKYTDK 880
LBC-ori  WIR-DNFTYNYNME-KEKYRITQYHHTHVRLKDLFPSRKIVKLEGYEALLAMMLDRFNNI 892
LBClus4  WIR-DNFTYNYNME-KEKYKITQYHHTHVRLKDLFPSRKVVKLTGYEALLAMMLDRFNNI 892
```

```
          : *  .:.*:**:  * . :*:*:**.*******: .:.:*.: *:** *. :**:::

LBCbarr2  AITHATFITYLRALPVREARLFIKLVIKYGSIDQEWLKEEGTVAKQIQGSTDFDLSKIFE 940
LBC-ori   ESTHVTFFTYLRALPDREKEVFISLVLNYNGLGREWLKSEGVRAKQAQGTVKYDMSKLFE 952
LBClus4   ESTHVTFFTYLRALPDQEREIFISLILNYNGLGREWLKSEGVRAKQAQGTVKYDMSKLFE 952
            **.**:******* :* .:**.*:::*..:.:****.**. *** **:..:*:**:**

LBCbarr2  LNVLHNRVETTVDWNEEREHRTHPDTVNISYPDVYTKCRELFELAKQEGKSPIKMDWNKY 1000
LBC-ori   LNVLENGVDEEVDWEKEKRNRSDIKTVNISYAKVLEHCRELFIMARAEGKRPMRMKWQEY 1012
LBClus4   LNVLENRVDEEVDWEKEKRNRTNIKTVDISYAKVLEHCRELFIMARAEGKRPMRMKWQEY 1012
          ****.* *:  ***::*:.:*:. .**:*** .*  :***** :*: *** *::*.*::*

LBCbarr2  WMQRASIMPSGAVHSQHSELDKYVRKLPREVKNKKGLAASLPNIDQRYFLSKIPSIEAYV 1060
LBC-ori   WRQRAVIMPGGSVHSQHPVEQDVIRVLPREIRSKKGVASVMPYKEQKYFTSRRPEIHAYT 1072
LBClus4   WRQRAVVMPGGSVHSQHPAEQDVIKILPREIKSKKGLASVMSYKEQKYFTSRRPEIHAYT 1072
          * *** :**.*:*****    :. :: ****::.***:*: :    :*:** *: *.*.**.
            ►——3——◄
LBCbarr2  STKYEWGKVRALYGCDFSSHVNADFGLLNCEDTFPHFIPTGPQATEGYVKTLLKTAKYNV 1120
LBC-ori   STKYEWGKVRALYGCDFSSHTMADFGLLQCEDTFPGFVPTGSYANEDYVRTRIAGTHSLI 1132
LBClus4   STKYEWGKVRALYGCDFSSHTMADFGLLQCEDTFPGFVPTGSYANEDYVRTRIAGTHSLI 1132
          ********************. ******:****** *:***  *.*.**:* :  ::  :
              ►—A——◄
LBCbarr2  PFCYDYDDFNSQHSKSSMQAVIDAWINTYFDDLTEDQIASALWTRESVGEMFVNDVKNSV 1180
LBC-ori   PFCYDFDDFNSQHSKEAMQAVIDAWISVYHDKLTDDQIEAAKWTRNSVDRMVAHQPNTGE 1192
LBClus4   PFCYDFDDFNSQHSKEAMQAVIDAWISVYHDKLTDDQVEAAKWTRNSVDRMIAHQTNTGE 1192
          *****:*********.:*********..*.*.**:**: :* ***:**..*..:: :..
                   ►———B——◄                       ►C◄
LBCbarr2  RYEAMGTLFSGWRLTTFINTALNYAYLAKAGINKLTNISIHNGDDVYAGTRNLKDIARLL 1240
LBC-ori   TYDVKGTLFSGWRLTTFFNTALNYCYLANAGINSLVPTSLHNGDDVFAGIRTIADGISLI 1252
LBClus4   TYDVKGTLFSGWRLTTFINTALNYCYLANAGINSLVPTSLHNGDDVFAGIRTIADGISLI 1252
           *:. ************:******.***:****.*.  *:******:** *.: *   *:
                               ► D ◄
LBCbarr2  KNSKQMGIRANTTKMSIGTIAEFLRVDMRAEKPTSAQYLTRGISTFVHGRIESEAPVGYR 1300
LBC-ori   KNAAATGVRANTTKMNIGTIAEFLRVDMRAKNSTGSQYLTRGIATFTHSRVESDAPLTLR 1312
LBClus4   RNAAATGVRANTIKMNIGTIAEFLRVDMRAKDSTGSQYLTRGVATFTHSRVESDAPLTLR 1312
          :*:    *:**** **.**************:. *.:******::**.*.*:**:**:  *

LBCbarr2  AMVSAYKTRYDEVVERGGNAQRLRHLYRKQLFFARRKFDVSEEMQEKLLKTHVQAGGLSV 1360
LBC-ori   NLVSAYKTRYDEILARGASIDNMKPLYRKQLFFARKLFNVEKDIVDNLITMDISCGGLQE 1372
LBClus4   NLVSAYKTRYDEIIARGANVDDMKPLYRKQLFFARKLFNVEKGIIDNLITMDISCGGLQD 1372
          :**********:: **.. : :: **********: *:*.: : ::*:. .:..***.

LBCbarr2  NGKIGNYTLEDDSLAYAD--IDVNEISSLLQPGVKDYVTSLKSLYPEIREFITEKTVRRT 1418
LBC-ori   KGRVSEMVLQEVDIENIDSYRKTRMIAKLIDKGVGDYTAFLKTNFSEIADAITRETRVES 1432
LBClus4   KGKVSEVVLQEVDIENIDSYKKTRMIAKLIDKGVGDYTAFLKTNFSEIADAITKKHVVES 1432
          :*::.: .*:: .:    *    ... *:.*:: ** **.: **: : ** : **.:   .:

LBCbarr2  LMKAFNINKNTICIKPASSKAIYNQIALRGAWNDDSNFRIFNRVRQGVSNVIGVLGKLSN 1478
LBC-ori   VTKAYNVKKKTVVRAFRDLSAAYHERAVRHAWKGMSGLHIVNRIRMGVSNLVMVVSKINP 1492
LBClus4   LTKAYNVKKKTVRSAFRDLSAAYHERAVRHAWKRMSGLHIVNRVRMGVSNLVMVVSKINP 1492
          : **:*::*:*:      . .* *:: *:* **:  *.::*.**:* ****:: *:.*:.

LBCbarr2  AHAKVLSETNDPIKWLSIYIT 1499
LBC-ori   AKANVLAKSGDPTKWLAVLT- 1512
LBClus4   AKANVLAKSGDPTKWLAVLT- 1512
          *:*:**:::.** ***::
```

Figure S2. Comparison of the amino acid sequences of Gag-Pol encoded by ScV-LBC1-original, ScV-LBClus4, and TdV-LBCbarr2. The separation between Gag and Pol domains is indicated (Gag ◄► Pol). The H156 residue required for 5´cap-snatching is black shaded. The four crucial residues for cap recognition (Tyr-152, Asn-154, Tyr-452 and Trp-545) are grey shaded. The highly conserved RdRp domain located in the central third of Pol is underlined, the five consensus motifs (3, A, B, C, and D) conserved in RNA-dependent RNA polymerases from Totiviruses are indicated above the sequence, and the conserved amino acids for each motif are grey shaded. Asterisks (*) indicate identical amino acids; colons (:) and single dots (.) indicate conserved and semi-conserved amino acids, respectively.

TdV-LBCbarr1-EX1180	100	94	52	52	52	52	52	52
TdV-LBCbarr2-EX1257	94	100	51	52	52	52	52	52
ScV-LBClus4-EX229	52	51	100	89	88	87	89	89
ScV-LBC2-S3920	52	52	89	100	93	94	94	94
ScV-LBC-original	52	52	88	93	100	95	95	95
ScV-LBC2-EX1125	52	52	89	94	95	100	100	100
ScV-LBClusA-EX1160	52	52	89	94	95	100	100	100
ScV-LBC1-EX231	52	52	89	94	95	100	100	100

Colors: ***T. delbrueckii* cluster** *S. cerevisiae* cluster

Figure S3. Percentage identity matrix for canonical V-LBC nucleotide sequences. Each value was rounded to the nearest whole number.

TdV-LBCbarr1-EX1180	100	99	64	66	66	66	66	66
TdV-LBCbarr2-EX1257	99	100	64	66	66	66	66	66
ScV-LBClus4-EX229	64	64	100	97	97	97	97	97
ScV-LBC2-S3920	66	65	97	100	98	98	98	98
ScV-LBC-original	65	65	97	98	100	99	99	99
ScV-LBClusA-EX1160	66	66	97	98	99	100	100	100
ScV-LBC1-EX231	66	66	97	98	99	100	100	100
ScV-LBC2-EX1125	66	66	97	98	99	100	100	100

Colors: ***T. delbrueckii* cluster** *S. cerevisiae* cluster

Figure S4. Percentage identity matrix for highly-conserved RdRp-domain amino-acid sequences. Each value was rounded to the nearest whole number.

TdV-LBCbarr1-EX1180	100	99	37	37	37	37	37	37
TdV-LBCbarr2-EX1257	99	100	37	37	37	37	37	37
ScV-LBClus4-EX229	37	37	100	98	97	97	97	97
ScV-LBC1-original	37	37	98	100	99	99	99	98
ScV-LBClusA-EX1160	37	37	97	99	100	100	100	98
ScV-LBC1-EX231	37	37	97	99	100	100	100	98
ScV-LBC2-EX1125	37	37	97	99	100	100	100	98
ScV-LBC2-S3920	37	37	97	98	98	98	98	100

Colors: ***T. delbrueckii* cluster** *S. cerevisiae* cluster

Figure S5. Percentage identity matrix for Gag amino-acid sequences of LBC viruses. Each value was rounded to the nearest whole number.
